# Adipose-derived stromal/stem cells are verified to be potential seed candidates for bio-root regeneration in three-dimensional culture

**DOI:** 10.1186/s13287-022-02907-y

**Published:** 2022-06-03

**Authors:** Yu Yuan, Xiaonan Zhang, Yuzhen Zhan, Song Tang, Pingmeng Deng, Zhenxiang Wang, Jie Li

**Affiliations:** 1 College of Stomatology, Chongqing Medical University, 426# Songshibei Road, Yubei District, Chongqing, 401147 People’s Republic of China; 2 Chongqing Key Laboratory of Oral Diseases and Biomedical Sciences, Chongqing, People’s Republic of China; 3 Chongqing Municipal Key Laboratory of Oral Biomedical Engineering of Higher Education, Chongqing, People’s Republic of China

**Keywords:** Adipose-derived stromal/stem cells, Dental follicle cells, Stem cells from human exfoliated deciduous teeth, Porcine treated cellular dentin matrix, Bio-root regeneration

## Abstract

**Background:**

Bio-root regeneration is a promising treatment for tooth loss. It has been reported that dental-derived stem cells are effective seed cells for bio-root construction, but further applications are limited by their few sources. Human adipose tissues have a wide range of sources and numerous studies have confirmed the ability of adipose-derived stromal/stem cells (ASCs) in regenerative medicine. In the current study, the odontogenic capacities of ASCs were compared with dental-derived stem cells including dental follicle cells (DFCs), and stem cells from human exfoliated deciduous teeth (SHEDs).

**Methods:**

The biological characteristics of ASCs, DFCs, and SHEDs were explored in vitro. Two-dimensional (2D) and three-dimensional (3D) cultures were compared in vitro. Odontogenic characteristics of porcine-treated dentin matrix (pTDM) induced cells under a 3D microenvironment in vitro were compared. The complexes (cell/pTDM) were transplanted subcutaneously into nude mice to verify regenerative potential. RNA sequencing (RNA-seq) was used to explore molecular mechanisms of different seed cells in bio-root regeneration.

**Results:**

3D culture was more efficient in constructing bio-root complexes. ASCs exhibited good biological characteristics similar to dental-derived stem cells in vitro*.* Besides, pTDM induced ASCs presented odontogenic ability similar to dental-derived stem cells. Furthermore, 3D cultured ASCs/pTDM complex promoted regeneration of dentin-like, pulp-like, and periodontal fiber-like tissues in vivo. Analysis indicated that PI3K-Akt, VEGF signaling pathways may play key roles in the process of inducing ASCs odontogenic differentiation by pTDM.

**Conclusions:**

ASCs are potential seed cells for pTDM-induced bio-root regeneration, providing a basis for further research and application.

**Graphical Abstract:**

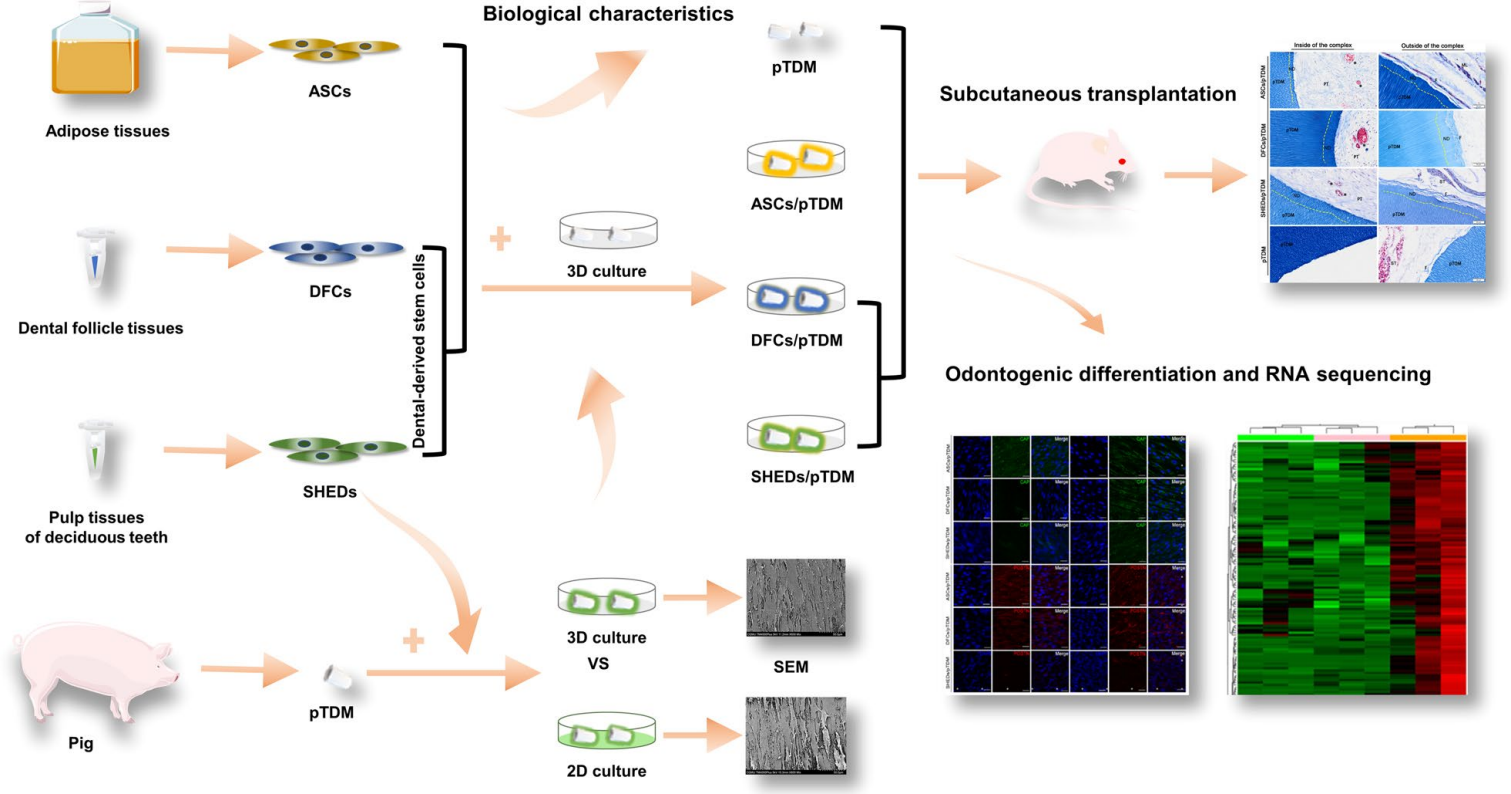

**Supplementary Information:**

The online version contains supplementary material available at 10.1186/s13287-022-02907-y.

## Introduction

Tooth loss is a common oral disease, caused by tooth decay, congenital malformations (tooth agenesis), trauma, periodontal diseases, or age-related changes [1]. It is associated with global health and economic burden [2]. Artificial dentures and metal implants are mainly used to repair these defects, however, they do not provide anatomical structure nor promote tissue regeneration [3]. Therefore, there is need to explore new therapeutic strategies for tooth loss. Tooth root is a multi-structure organ composed of soft tissues of dental pulp and periodontium, and mineralized tissues (dentin and cementum) [4]. Mineralized tissues are an integral part of the tooth and play key roles in maintaining tooth functions and supporting a natural or artificial crown [5]. Bio-root regeneration through stem cells restoration has been widely explored recently. Stem cells are combined with root-shaped scaffolds then implanted into the alveolar bone to form functional roots with root-like structure, biomechanical properties, and elements similar to those of natural teeth including periodontal ligament-like tissue and dentin like matrix [6]. Three elements are required for bio-root regeneration including scaffold materials, microenvironments, and seed cells [7].

Treated dentin matrix (TDM) from natural tooth tissue has been used as a biological scaffold for bioengineering tooth organs owing to its biological property of providing an odontogenic microenvironment [8, 9]. Several studies report the advantages of dental-derived stem cells combined with TDM in bio-root regeneration [10–14]. Although human TDM (hTDM) is widely used in the construction of bio-root, it is impractical to obtain enough sources. Due to its wide sources and similar shape to hTDM, porcine TDM (pTDM) is used as an alternative source for hTDM, therefore circumventing the limitation of shortage of allogeneic TDM [9, 13, 15]. Moreover, spatial characteristics of the cell culture microenvironment have a significant impact on the fate of stem cells [16]. Compared with the traditional two-dimensional (2D) culture, three-dimensional (3D) culture can more comprehensively simulate the natural environment of stem cells and provide more complete intercellular and intercellular matrix interactions [17, 18]. The 3D cultured cells maintained more consistent morphology and gene expression with those cells in natural tissues [19]. 3D culture methods include scaffold-free and scaffold-based systems, in which scaffolds include biological scaffolds and synthetic scaffolds [19–21]. TDM, as a biological scaffold material for bio-root construction, provides the mechanical shape of natural root [22], presents good biocompatibility and bioactivity, and releases abundant important proteins including DSPP, DMP1, COL-1, TGF-β1, which play key roles in tooth development, providing a natural odontogenic 3D induction microenvironment [8, 13, 15, 23].

Researchers have proved that dental follicle cells (DFCs) or stem cells from human exfoliated deciduous teeth (SHEDs) combined with TDM implanted in animals (nude mice, rats, rhesus) present effective regeneration of bio-root [10, 11, 13, 24]. In addition, complexes obtained from the combination of exosome-like vesicles derived from Hertwig's epithelial root sheath cells (ELVs-H1), dental papilla cells (DPCs), and TDM promote regeneration of dentin-pulp tissue [25]. Although these dental-derived stem cells show great potential in root tissues regeneration, limited tissue sources constrain further applications. Sources of the dental follicle and dental papilla to isolate DFCs, DPCs are limited owing to a growing number of people who miss the third molar for the evolution of human beings [14]. In addition, pulp tissues of deciduous teeth used to isolate SHEDs are tiny amounts because that root resorption of deciduous teeth is accompanied by pulp resorption in the process of permanent teeth eruption [26]. Moreover, the natural loss of deciduous teeth does not often occur in clinics [27]. Therefore, studies should explore new seed cells for bio-root regeneration to develop a new strategy for the treatment of tooth loss.

Preliminary studies by Zuk et al. reported the multipotency of adipose-derived stromal/stem cells (ASCs) for the first time [28, 29]. Adipose tissues are ubiquitous tissues that can be obtained in large amounts through minimally invasive surgery (liposuction). Moreover, adipose tissues contain abundant ASCs that are requisite for stem cells therapies [30]. Several studies have explored the application of ASCs in regenerative medicine and confirmed that transplanting ASCs into periodontal tissue defects in mouse or rat models can promote regeneration of cementum, periodontal ligament fibers, and periodontal blood vessels [31, 32]. In addition, ASCs can regenerate dentin, periodontal ligament, and alveolar bone structure in adult rabbits [33]. These properties of ASCs imply that they are effective candidates for bio-root regeneration.

The current study sought to determine the effect of ASCs on bio-root regeneration and to explore a new and abundant cell source for the construction and clinical application of bio-root. pTDM was used as scaffold material in the current study. DFCs, and SHEDs were selected as control groups to represent dental-derived stem cells for comparison of the biological characteristics of DFCs, SHEDs, and ASCs in the present study. The odontogenic differentiation ability of the three types of cells cultured under a 3D microenvironment was explored in vitro. To further compare the bio-root regenerative potential of ASCs, DFCs, and SHEDs in vivo, cell/pTDM complexes were subcutaneously transplanted in nude mice. RNA sequencing (RNA-seq) was performed to explore molecular mechanisms underlying the role of different seed cells in bio-root regeneration.

## Materials and methods

### Cell culture

#### Primary culture of DFCs and SHEDs

Impacted third molars of 16–25-year-old healthy young patients and deciduous teeth of 6–12-year-old children extracted for clinical reasons were obtained for cell isolation. The study was approved by the Ethics Committee of College of Stomatology, Chongqing Medical University. Written informed consent was obtained from all participants and guardians representing the children in the current study. Dental follicles of impacted third molars and pulp tissues of deciduous teeth were dissected and rinsed with sterile phosphate-buffered saline (PBS, Solarbio, Beijing, China). The tissues were cut into 1 × 1 mm sections and digested with 3% type I collagenase (Sigma-Aldrich, St. Louis, MO, USA). Tissue sections were cultured in α-minimum essential medium (α-MEM, HyClone, Logan, UT, USA) supplemented with 10% fetal bovine serum (FBS, Gemini Bio-Products, Woodland, CA, USA) under 5% CO_2_ humidified incubator at 37 °C.

#### Primary culture of ASCs

Subcutaneous adipose tissue (stored in syringes) from the abdomen or thighs of women aged 20–27 who underwent liposuction for cosmetic reasons was collected, stored in an icebox, and transferred to the laboratory. All experiments were approved by the Ethics Committee of College of Stomatology, Chongqing Medical University, and written informed consent was obtained from all tissue donors in this study. The adipose tissues were transferred to a 50 mL centrifuge tube, and the same volume of PBS was added to suck out the liquid containing blood at the bottom of the centrifuge tube under biosafety cabinets following our published protocol [34]. Tissue samples were washed three times, and then digested with 0.1% collagenase solution of the same volume as adipose tissue and cultured in α-MEM supplemented with 10% FBS under 5% CO_2_ humidified incubator at 37 °C.

### Colony formation assay

1 mL cell suspension of ASCs, DFCs, and SHEDs were seeded in separate 100 mm diameter culture dishes, which was equivalent to 1000 cells per dish. The three types of cells were fixed with 4% paraformaldehyde (Beyotime, Shanghai, China) after 10 days of culture. Cells were then stained with crystal violet (Beyotime, Shanghai, China), and washed 1–2 times after staining. Colony formation was observed and colonies were counted. The experiment was performed with at least three technical and biological replicates.

### Cell proliferation assay

The proliferation of ASCs, DFCs, and SHEDs was explored using Cell Counting Kit-8 (CCK-8, Dojindo, Kumamoto, Japan). Cell suspension (1000 cells/well) was dispensed in 96-well plates. The medium was changed to 100 μL α-MEM containing 10% FBS after 24 h and 10 μL CCK-8 solution was added and cells were incubated for 4 h. The absorbance was measured at 450 nm using a multimode flat-panel reader (PerkinElmer, Waltham, Massachusetts, USA), and recorded as the value for day 0. Absorbance was further measured every two days until day 7. At least three technical replicates and biological replicates were performed.

### Flow cytometry analysis

ASCs, DFCs, and SHEDs were digested into single-cell suspension by treatment with trypsin. Samples were then incubated with Fluorescein Isothiocyanate (FITC) binding antibodies against CD14, CD19, CD45, CD90, CD105, CD146, and phycoerythrin (PE) binding antibody against CD73 to determine the expression levels of cell surface markers. All antibodies were purchased from BD Biosciences (CA, USA). After incubation, samples were washed 3 times with PBS. Cells were then suspended in PBS for flow cytometry analysis using a flow cytometer (BD Biosciences, CA, USA). The experiment was performed with at least three technical and biological replicates.

### Cell cycle analysis

ASCs, DFCs, and SHEDs were adjusted to single-cell suspension by trypsin and the cell cycle was explored using the Cell cycle Kit (Beyotime, Shanghai, China). Cold PBS was added to suspend the cells then cells were centrifuged and precipitated. The supernatant was then retrieved. Cells were fixed with 1 mL of precooled 70% ethanol at 4 °C for 30 min and then centrifuged to precipitate the cells using precooled PBS. 0.5 mL propidium iodide staining solution was added to each tube containing cells samples. Samples were suspended slowly, then completely suspended and incubated for 30 min at 37 °C under dark conditions. Flow cytometry was performed to detect the red fluorescence at the excitation wavelength of 488 nm. Further, light scattering was detected using CytoFLEX (Beckman Coulter, Inc., Brea, CA, USA). The experiment comprised three technical replicates and biological replicates.

### Cell apoptosis

Cell apoptosis Kit (Beyotime, Shanghai, China) was used to explore the apoptosis rates of ASCs, DFCs, and SHEDs after adjustment to single-cell suspension by digestion using trypsin. Cells at a density of 5–10 × 10^5^ were used in this assay. Cells were centrifuged, the supernatant was discarded and 195 μL annexin V-FITC binding solution was added to suspend the samples. Further, 5 μL annexin V-FITC and 10 μL propidium iodide staining solution were added to the suspension then mixed gently. Cells were incubated at room temperature (20–25 °C) under dark conditions for 10–20 min then placed in an ice bath. Detection by CytoFLEX (Beckman Coulter, Inc., Brea, CA, USA) indicates that annexin V-FITC has green fluorescence and propidium iodide (PI) has red fluorescence. At least three technical and biological replicates were performed.

### SA-β-gal staining

Senescence-associated β-galactosidase (SA-β-gal) staining was performed using the SA-β-gal staining kit (Beyotime, Shanghai, China). ASCs, DFCs, and SHEDs at passage 13 were harvested. Cells were washed three times with PBS and fixed with the SA-β-gal staining agent for 15 min at room temperature then stained with the SA-β-gal staining solution overnight at 37 °C. The number of blue cells (positive staining for SA-β-gal) in five different fields of view was randomly selected for each sample from three independent experiments under a phase-contrast inversion microscope (Olympus, Tokyo, Japan). The percentage of positive cells from the three independent experiments was then calculated. The assay comprised at least three technical replicates and biological replicates.

### Adipogenic differentiation

ASCs, DFCs, SHEDs were adjusted to a density of 1 × 10^5^/mL then seeded in 6-well plates. The culture medium was replaced with α-MEM supplemented with 10% FBS (complete medium) containing 0.5 mM isobutylmethylxanthine (IBMX, Sigma-Aldrich, St. Louis, MO, USA), 10 uM insulin (Sigma-Aldrich, St. Louis, MO, USA), 200 uM indomethacin (Sigma-Aldrich, St. Louis, MO, USA), and 1 uM dexamethasone (Sigma-Aldrich, St. Louis, MO, USA) after cells reached 80–90% confluency. The medium was changed every 2–3 days. After 7 days of culture, cells were fixed with 4% paraformaldehyde for 15 min and further washed with 60% isopropanol for 1 min and 3 times with PBS washing, then observed and photographed under a phase-contrast inverted microscope (Olympus, Tokyo, Japan). At least three technical and biological replicates were performed.

### Osteogenic differentiation

Suspension of ASCs, DFCs, and SHEDs containing 1 × 10^5^ cells was added to 6-well plates. When 80–90% fusion was achieved, the cells were cultured in a medium comprising 10% FBS, 100 μM dexamethasone (Sigma-Aldrich, St. Louis, MO, USA), 10 mM L-glycerophosphate (Sigma-Aldrich, St. Louis, MO, USA), and 50 μg/mL ascorbic acid (Sigma-Aldrich, St. Louis, MO, USA). The culture medium was changed every 2–3 days. After 3 weeks of culture, cells were fixed with 4% polyformaldehyde for 15 min, then washed twice with PBS. 1% alizarin red solution (Sigma-Aldrich, St. Louis, MO, USA) was used to incubate with cells at room temperature for 30 min, and then cells were washed twice with PBS. Observation and photography were performed under a phase-contrast inversion microscope (Olympus, Tokyo, Japan). Calcified nodules were eluted with 10% cetylpyridinium chloride monohydrate (Sigma-Aldrich, St. Louis, MO, USA) in 10 mM sodium phosphate. Absorbance was measured at 540 nm using a multimode flat-panel reader (PerkinElmer, Waltham, Massachusetts, USA) to determine the concentration of calcium. The assay comprised at least three technical replicates and biological replicates.

### Neurogenic differentiation

Densities of ASCs, DFCs, SHEDs cells were adjusted to 1 × 10^4^/mL and then seeded in 24-well plates. After achieving 70% confluence, cells were cultured in a medium comprising 10% FBS, 200 μM butylated hydroxyanisole (Sigma-Aldrich, St. Louis, MO, USA), 1 mM hydrocortisone (Solarbio, Beijing, China), 25 mM KCl (Solarbio, Beijing, China), 2 mM valproic acid (Sigma-Aldrich, St. Louis, MO, USA), 10 μM forskolin (Solarbio, Beijing, China), 5 μg/mL insulin (Sigma-Aldrich, St. Louis, MO, USA), 2 mM L-Glutamine (Sigma-Aldrich, St. Louis, MO, USA) and 2% dimethyl sulfoxide (Sigma-Aldrich, St. Louis, MO, USA) for 14 days. The expression of β-III tubulin (1:100 dilution, Santa Cruz, Santa Cruz Biotechnology, CA, USA) was then detected by immunofluorescence staining. Images were obtained and analyzed under a fluorescence microscope (Olympus, Tokyo, Japan). The experiment was performed in at least three technical and biological replicates.

### Detection of pluripotency markers and embryonic layers markers

ASCs, DFCs, and SHEDs at passages 1, 3, 5, and 7 were harvested for extraction of total RNA using RNAiso Plus (Takara Biomedical Technology, Beijing, China) according to the manufacturer’s protocol. Prime-Script™ RT reagent kit (Takara Biomedical Technology, Beijing, China) was used to reverse transcribe the RNA into cDNA. Quantitative real-time PCR (RT-qPCR) was performed using SYBR Premix (QIAGEN, Hilden, Germany) to quantify the expression levels of *Nanog*, *Sox2*, *PDGFRα*, and *β-III tubulin*. At least three technical and biological replicates were performed.

### Preparation of pTDM

Deciduous incisors were obtained from slaughtered pigs in the morning following a method reported previously [10]. The pulp tissue, anterior dentin, and periodontal tissue were completely removed using a turbine. pTDM was formed a cone with a length of about 6–7 mm and a diameter of about 3–5 mm. The matrix was mechanically cleaned in deionized water using an ultrasonic cleaning machine and then treated with 17% ethylenediaminetetraacetic acid (EDTA, Sigma-Aldrich, St. Louis, MO, USA) for 20 min, 10% EDTA for 20 min, and 5% EDTA for 20 min. Samples were stored in Penicillin–Streptomycin solution (HyClone, Logan, UT, USA) for 24 h, then finally stored in a − 80 °C refrigerator. Morphology and histology of pTDM were observed using scanning electron microscopy (SEM, TM4000PLUS II, Hitachi, Tokyo, Japan).

### Comparison of 2D and 3D cultures

In this study, pTDM, a biological scaffold with a 3D structure, was combined with a special culture plate to construct a 3D culture system. In that SHEDs have been proved to be bio-root regeneration seed cells in the previous study [14], it was used to evaluate the efficiency of the 3D culture system.

5 × 10^5^ and 3 × 10^5^ SHEDs were seeded separately onto pTDM in traditional 6-well culture plates and ultra-low adhesion 6-well culture plates (Corning Incorporated, Corning, N.Y., USA). The complexes were harvested on the fourth day of culture and used in subsequent SEM, RT-qPCR, immunofluorescence analyses. Adherence of cells in 2D and 3D cultures within 24 h was observed under a phase-contrast inversion microscope (Olympus, Tokyo, Japan). The test was repeated at least three times including technical and biological replicates.

### Preparation of the ASCs/pTDM, DFCs/pTDM and SHEDs/pTDM complexes

ASCs, DFCs, and SHEDs at a density of 1 × 10^6^ cells were seeded into ultra-low adhesion 6-well culture plates containing pTDM scaffolds. The complexes of the three groups were harvested on the fourth and seventh day of culture for subsequent SEM, RT-qPCR, immunofluorescence and RNA-seq analyses, and animal experiments.

### SEM

SEM observations were performed to determine whether the dentin tubules on the surface of pTDM were completely exposed and to explore the attachment of ASCs, DFCs, and SHEDs on the surface of pTDM. Cell/pTDM complexes were cultured in vitro for 4 days, then the samples containing pTDM scaffolds and cell/pTDM complexes were washed 2 times with PBS and fixed in electron microscope fixative (2% glutaraldehyde, Beyotime, Shanghai, China). After dehydration using ethanol, the coatings were sputtered with gold, and samples were observed under a scanning electron microscope (SEM, TM4000PLUS II, Hitachi, Tokyo, Japan). At least three technical and biological replicates were performed.

### Comparison of Odontogenic differentiation of ASCs and dental-derived stem cells

ASCs/pTDM complex was used as the experimental group, whereas DFCs/pTDM, and SHEDs/pTDM complexes were selected as control groups, and cells were used as the blank control group. A high density of ASCs, DFCs, and SHEDs was covered with pTDM for 0, 4, and 7 days. Total RNA was extracted from cells of each group using RNAiso Plus according to the manufacturer’s protocol. Prime-Script™ RT reagent kit was used to reverse transcribe the RNA into cDNA. RT-qPCR was then performed using SYBR Premix to determine the expression levels of related genes, including *DSPP*, *OCN*, *COL-I*, *ALP*, *TGF-β1*, *POSTN*, *KDR*, *COMP*, *FGF18*, *COL16A1*, *MMP8*, *Gli2*, *RUNX2*, *VEGFA*, *VEGFB*, and *GAPDH*. The 2^−ΔΔCT^ method was used to determine the relative mRNA expression levels after normalization based on the GAPDH reference gene. RT-qPCR primer sequences are presented in Additional file 1: Table S1. The test comprised at least three technical and biological replicates.

Expression levels of odontogenic associated proteins were determined by immunofluorescence analysis. Cell/pTDM complexes cultured for 4 and 7 days in vitro were washed 2–3 times with cold PBS, then fixed with 4% paraformaldehyde for 10 min. Complexes were further treated with 0.1% Triton X100 (Sigma-Aldrich, St. Louis, MO, USA) for 5 min and blocked with 1% BSA (Sigma-Aldrich, St. Louis, MO, USA) for 30 min. Samples were incubated with primary antibodies against DMP-1 (1:100 dilution, Santa Cruz, Santa Cruz Biotechnology, CA, USA), CAP (1:100 dilution, Santa Cruz, Santa Cruz Biotechnology, CA, USA), and POSTN (1:100 dilution, Proteintech Group, Wuhan, China) at 4 °C under a moist chamber overnight. Samples were further incubated with secondary antibodies including goat anti-rabbit Alexa Fluor 555 (Bioss, Beijing, China) or goat anti-mouse Alexa Fluor 488 (Bioss, Beijing, China) horseradish peroxidase binding IgG antibody for 1 h and stained with DAPI (Beyotime, Shanghai, China) staining solution for 5 min. The protein expression levels were then determined by laser confocal scanning (Leica Optical, Wetzlar, Germany) and photographed. At least three technical and biological replicates were performed. The average fluorescence intensity of three fluorescence images with different visual fields in the same group was detected, and the specific protein expression was semi-quantitatively analyzed by ImageJ V1.8.0.112 software.

### Subcutaneous transplantation in nude mice

Animal experiments in the present study were approved by the Ethics Committee of College of Stomatology, Chongqing Medical University. Nude mice used in the experiment were purchased from Chongqing Tengxin Biotechnology Co., Ltd (Chongqing, China).

ASCs/pTDM complex was selected as the experimental group, whereas DFCs/pTDM and SHEDs/pTDM complexes were the control groups, and pTDM was the blank control group. To further explore the odontogenic ability of ASCs, DFCs, and SHEDs in vivo, cells/pTDM complexes (ASCs/pTDM, DFCs/pTDM, and SHEDs/pTDM) cultured for 4 days in vitro and pTDM were transplanted into the dorsal side of immunodeficient mice (8-week-old male, n = 6). The procedure was performed under general anesthesia using isoflurane. Two longitudinal subcutaneous incisions about 1 cm long were made on the back and two subcutaneous bags were created on the left and right sides of each incision to administer the implant as follows: ASCs/pTDM (n = 6), DFCs/pTDM (n = 6), SHEDs/pTDM (n = 6), pTDM alone (n = 6). Implants were removed 8 weeks after implantation and decalcified with 10% EDTA for 4 months. The specimens were embedded in paraffin, sectioned, and stained with hematoxylin–eosin (H&E, Solarbio, Beijing, China), and Masson’s Trichrome (Solarbio, Beijing, China). Immunohistochemistry was performed as described below. Sections from each group, including decalcified ASCs/pTDM complex (experimental group), DFCs/pTDM and SHEDs/pTDM complexes (control group), and pTDM scaffold (blank control group), were dewaxed, rehydrated, and incubated with primary antibodies (DSPP, DMP-1, CAP, TGF-β1; 1:100 dilution, Santa Cruz, Santa Cruz Biotechnology, CA, USA) and anti-mitochondria antibody (1:1000 dilution, Abcam, Cambridge, MA, UK) in a moist chamber at 4 °C overnight. PBS was used as the negative control of primary antibodies. Samples were further incubated with secondary antibodies at room temperature for 0.5–1 h. The slides were stained with diaminobenzidine (DAB, ZSJQ-BIO, Beijing, China) and observed under a microscope (Olympus, Tokyo, Japan).

### RNA sequencing

RNA integrity was determined using the RNA Nano 6000 Assay Kit of the Bioanalyzer 2100 system (Agilent Technologies, CA, USA). Total RNA was used as input material for RNA sample preparations. The cell/pTDM complexes (ASCs/pTDM, DFCs/pTDM, and SHEDs/pTDM) were cultured for 4 days in vitro (n = 9). The clustering of the index-coded samples was performed on a cBot Cluster Generation System using TruSeq PE Cluster Kit v3-cBot-HS (Illumina) according to the manufacturer’s instructions to explore the global expression profile of the complexes. After cluster generation, libraries were sequenced on an Illumina Nova seq platform and 150 bp paired-end reads were generated. Clean data (clean reads) were obtained by removing reads containing adapters, reads containing N base and low-quality reads from the raw data. Index of the reference genome was built using the Hisat2 v2.0.5 tool and paired-end clean reads were aligned to the reference genome using Hisat2 v2.0.5. FPKM of each gene was calculated based on the length of the gene and the reads count mapped to this gene. Differential expression analysis was performed using the DESeq2 package and genes with an adjusted *p* value < 0.05 in DESeq2 were selected as differentially expressed. Gene Ontology (GO) and KEGG enrichment analyses of differentially expressed genes were performed using the cluster Profiler R package, in which gene length bias was corrected. In addition, a total of 70 tooth development-related genes were screened from the transcriptome analysis of ASCs/pTDM, DFCs/pTDM, and SHEDs/pTDM complexes, and cluster analysis, GO and KEGG enrichment analyses, and PPI analysis were performed.

### Statistical analysis

All experiments in this study were performed in triplicates. Data were presented as mean ± standard error of the mean (SEM). One-way analysis of variance (ANOVA) and two-way ANOVA were performed to explore statistical differences. GraphPad Prism 8.0.2 software was used for statistical analysis and generation of figures and plots. P values < 0.05 were considered statistically significant.

## Results

### Biological characteristics of ASCs, DFCs, and SHEDs

**Fig. 1:**
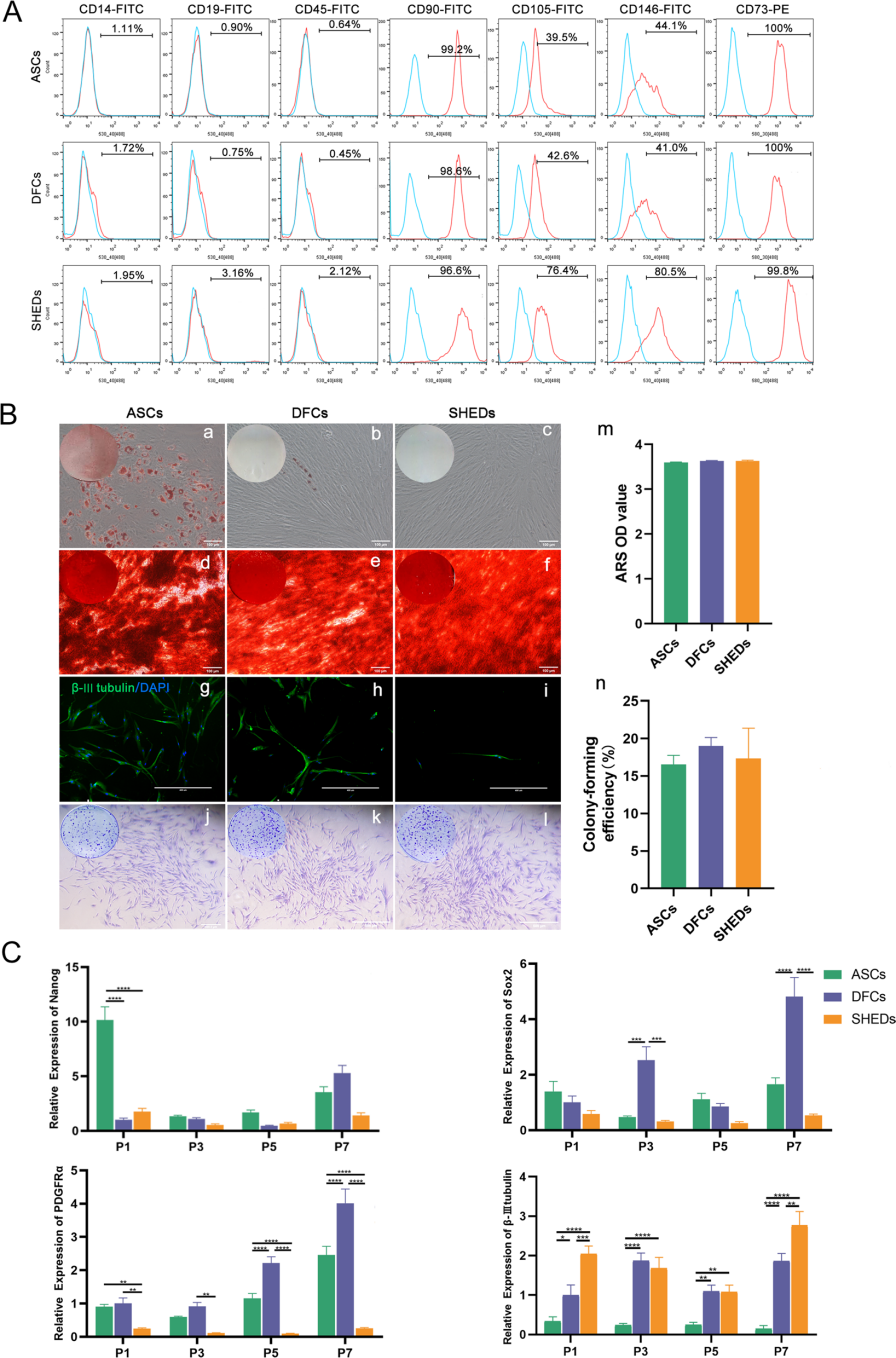
Biological characteristics and expression levels of pluripotency and embryonic layers markers of adipose-derived stromal/stem cells (ASCs), dental follicle cells (DFCs), and stem cells from human exfoliated deciduous teeth (SHEDs). (**A**) CD73, CD90, CD105, and CD146 were expressed in ASCs, DFCs, and SHEDs, however, these cells did not express CD14, CD19, and CD45. (**B** a, b and c) Oil red O staining of lipid droplets in ASCs, DFCs, and SHEDs after adipogenic induction. (**B** d, e, and f) Mineralized nodules were observed in the three cell types after 21 days of osteogenic induction. (**B** m) The findings showed no statistical difference in the number of mineralized nodules among ASCs, DFCs, and SHEDs groups. (**B** g, h, and i) Positive staining of β-III tubulin was observed in ASCs, DFCs, and SHEDs after nerve induction. (**B** j, k, and l) Crystal violet staining of colony-forming unit fibroblasts showed that ASCs, DFCs, and SHEDs cell types exhibited high colony-forming ability. (**B** n) There was no significant difference in colony formation efficiency among the three groups. (**C**) *Nanog*, *Sox2*, *β-III tubulin*, and *PDGFRα* expression levels in ASCs, DFCs, and SHEDs were determined by RT-qPCR. Scale bars = 100 μm (B a, b, c, d, e, and f), scale bars = 400 μm (**B** g, h, and i), scale bars = 500 μm (**B** j, k, and l). *p < 0.05, **p < 0.01, ***p < 0.001, ****p < 0.0001

Primary cells of ASCs, DFCs, and SHEDs harvested from adipose tissues, dental follicle tissues, and pulp tissues of deciduous teeth showed similar morphology and had fibroblast-like spindle shape. ASCs, DFCs, and SHEDs were collected at passage 4 for flow cytometric analysis. The findings showed that the three types of cells had similar immunophenotype profiles. The three cell types expressed mesenchymal stem cell markers including CD73, CD90, CD105, and CD146, however, they did not express monocyte marker, CD14, B cell marker, CD19, and leukocyte marker, CD45 (Fig. 1A). Further analysis was performed to explore the multi-directional differentiation ability of the three kinds of cells. Oil Red O staining was used to explore adipogenesis and the findings showed that ASCs formed significantly more lipid droplets compared with DFCs and SHEDs. Lipid droplets formed by DFCs were slightly more compared with those formed by SHEDs (Fig. 1B a, b, and c). Results of osteogenesis by alizarin red staining showed that a large number of calcium nodules was formed in the three kinds of cells (Fig. 1B d, e, and f), and the number of calcium nodules was not significantly different among the three cell types (Fig. 1B m). The findings of neurogenesis showed that the three kinds of cells differentiated into neuron-like cells, and all expressed the neural cell marker, β-III tubulin as shown by immunofluorescence staining (Fig. 1B g, h, and i). Crystal violet staining displayed that the three cell types effectively formed colonies from a single cell (Fig. 1B j, k, and l). Analysis revealed that colony-forming efficiency of DFCs was higher compared with that of SHEDs, and colony-forming efficiency of SHEDs was higher compared with that of ASCs, however, the differences among them were not statistically significant (Fig. 1B n). The findings showed that the contents of DNA in ASCs, DFCs, and SHEDs in the G1 phase were 90.10 ± 6.80%, 75.40 ± 5% and 79.10 ± 8.50%, respectively. The levels in the G2 phase were 5.20 ± 1.10%, 5.40 ± 5.10%, and 8.20 ± 2.80%, respectively. The levels in the S phase were 6.90 ± 2.50%,19.30 ± 2.70%, and 12.70 ± 10.2% respectively. Notably, the difference in DNA content among the three cell types was not statistically significant (Additional file 2: Figure S1A a, b, c, d, e, and f). Annexin V and PI double staining apoptosis kit was used to determine cell apoptosis rate for the three cell types. Percentages of apoptotic cells of ASCs, DFCs, and SHEDs were 9.50 ± 6.20%, 5.20 ± 2.10%, and 8.50 ± 0.90%, respectively, and analysis showed that the differences were not statistically significant (Additional file 2: Figure S1B a, b, c, and d). CCK-8 assay was performed to determine the proliferation ability of the three cell types on days 0, 3, 5, and 7 by measuring the absorbance at 450 nm. The results demonstrated that the proliferation rate of ASCs was between that of DFCs and SHEDs, and the difference in proliferation rate among the three cell types was statistically significant (Additional file 2: Figure S1C). Senescent cells are mainly characterized by increased activity of β-galactosidase [35]. Therefore, the senescence level of ASCs, DFCs, and SHEDs cells was explored at passage 13 (P13) using a kit according to the manufacturer's instructions. The findings showed no significant senescence cells at P13 for the three cell types (Additional file 2: Figure S1D).

### Pluripotency markers and embryonic layers markers of ASCs, DFCs, and SHEDs

*Nanog* and *Sox2* play important roles in stem cell self-renewal, maintenance of stem cell pluripotency, and reprogramming of somatic cells into pluripotent stem cells [36]. RT-qPCR analysis presented that *Nanog* and *Sox2* were expressed from passage 1 (P1) to passage 7 (P7) for the three cell types (Fig. 1C). The expression of *Nanog* in ASCs was significantly higher compared with the levels in DFCs and SHEDs at P1. However, the analysis showed no differences in the expression of *Nanog* in ASCs at passage 3 (P3), passage 5 (P5), passage 7 (P7) compared with the expression in DFCs and SHEDs (Fig. 1C). ASCs displayed a similar expression of *Sox2* to that of SHEDs at P1, P3, P5, P7. Moreover, the expression of *Sox2* was significantly lower compared with the levels of *Sox2* in DFCs at P3 and P7 (Fig. 1C). *PDGFRα* is a mesodermal marker [37] and the findings showed that it was expressed in the three cell types at P1-P7 (Fig. 1C). The expression of *PDGFRα* in ASCs was higher compared with the expression in SHEDs. The expression of *PDGFRα* in DFCs was higher compared with the expression level in ASCs at P1, P5, P7 (Fig. 1C). The findings showed that *β-III tubulin*, an ectodermal marker [38] was expressed at P1-P7 of the three cell types (Fig. 1C). The expression of *β-III tubulin* in ASCs at P1-P7 was lower compared with the levels in DFCs and SHEDs (Fig. 1C).

### 3D culture was more effective for odontogenic differentiation

**Fig. 2:**
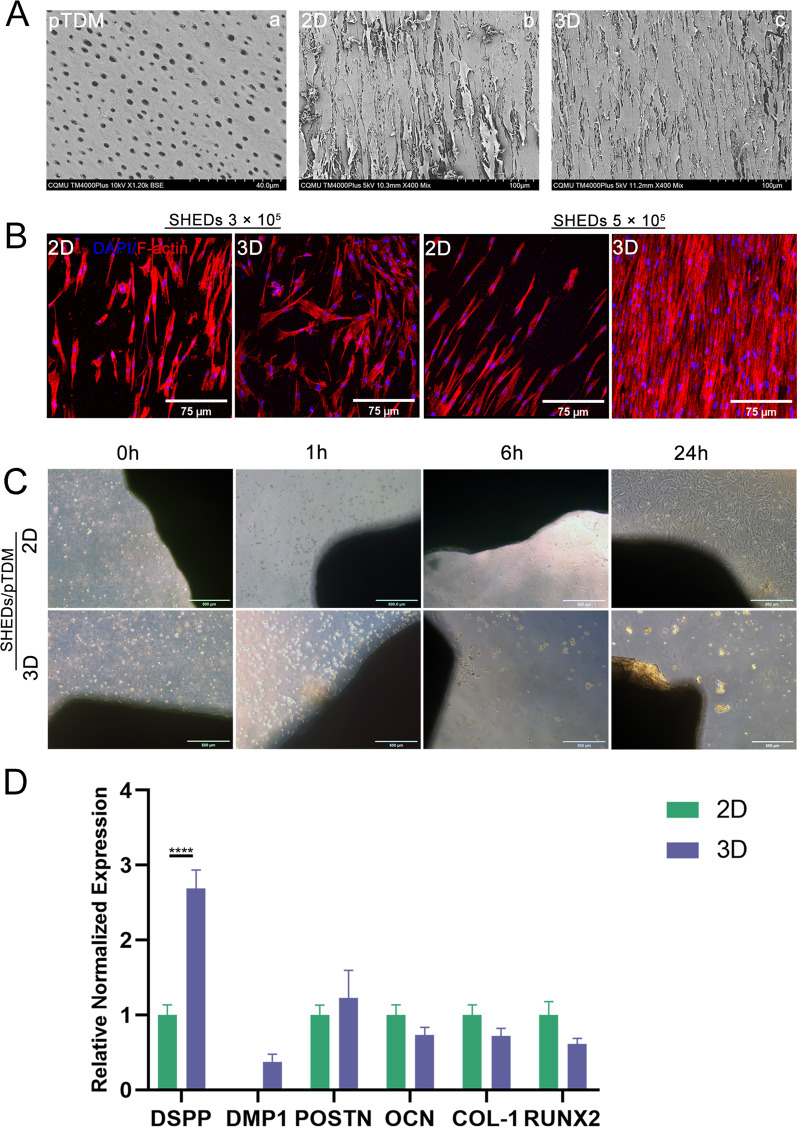
Comparison of two-dimensional (2D) and three-dimensional (3D) cultures. (**A** a) Scanning electron microscopy (SEM) analysis showed that porcine treated dentin matrix (pTDM) fully exposed dentinal tubules. (**A** b, and c) More cells were observed on the surface of SHEDs/pTDM complex cultured in 3D conditions compared with those cultured under 2D conditions detected by SEM. (**B**) Positive staining of phalloidin showed that 5 × 10^5^ SHEDs combined with pTDM cultured in 3D conditions exhibited higher cell adherence to the surface of pTDM compared with 2D culture. (**C**) Changes in SHEDs/pTDM complexes cultured in 2D and 3D conditions from 0 to 24 h. (**D**) Expression levels of odontogenesis-related genes (*DSPP*, *DMP1*, *POSTN*, *OCN*, *COL-1*, and *RUNX2*) in SHEDs/pTDM complex cultured in 3D and 2D conditions. Scale bar = 40 μm (**A** a), scale bars = 100 μm (**A** b, and c), scale bars = 75 μm (**B**), scale bars = 500 μm (**C**). **** p < 0.0001

SEM analysis showed that the dentin tubules of pTDM were fully exposed (Fig. 2A a). Immunofluorescence analysis of the phalloidin revealed that the number of cells on the surface of pTDM in 3D culture was higher compared with that in 2D culture, mainly for the group of 5 × 10^5^ SHEDs (Fig. 2B). Therefore, this number of cells was selected for combination with pTDM and the complexes were cultured for 4 days in 3D and 2D conditions then SEM analysis was performed. The findings showed that there more cells were attached to the surface of pTDM cultured under the 3D microenvironment compared with those cultured under the 2D microenvironment (Fig. 2A b, and c). In addition, complexes of 5 × 10^5^ SHEDs and pTDM were cultured under 3D and 2D microenvironments. The cells were observed and photographed under a microscope at 0 h, 1 h, 6 h, and 24 h. The findings showed that most of the cells cultured under the 2D microenvironment had completely adhered to the petri dish bottom after 24 h, whereas few of the cells cultured under the 3D microenvironment were suspended in the culture medium in a spherical shape (Fig. 2C). These results indicate that more cells were attached to the surface of pTDM under 3D culture. Moreover, RT-qPCR analysis was performed for complexes of pTDM and 5 × 10^5^ SHEDs under 2D culture and 3D culture conditions. The findings showed that SHEDs/pTDM complex cultured under the 3D microenvironment expressed higher levels of odontogenic markers, mainly *DSPP*. The expression of *DMP1* and *POSTN* in 3D culture was higher compared with that in 2D culture, however, the expression levels were not statistically different. The expression levels of osteogenesis-related genes including *OCN*, *COL-1*, and *RUNX2*, were not statistically different under 2D and 3D cultures (Fig. 2D).

### Odontogenic differentiation ability of ASCs, DFCs, and SHEDs in vitro

**Fig. 3:**
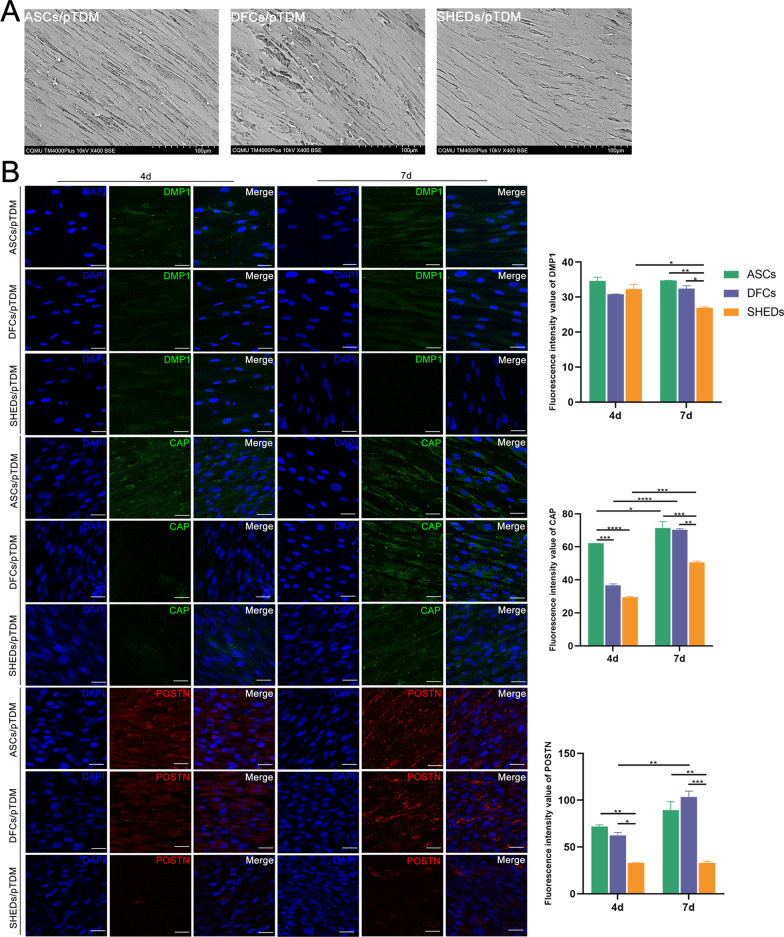
SEM analysis and expression levels of odontogenesis-related proteins in cell/pTDM complexes. (**A**) Cell adhesion on cell/pTDM complexes (ASCs/pTDM, DFCs/pTDM and SHEDs/pTDM) surfaces was detected by SEM after 4 days of pTDM induction after culturing in 3D conditions. (**B**) Immunofluorescence was performed to determine expression levels of odontogenesis-related proteins in ASCs/pTDM, DFCs/pTDM, and SHEDs/pTDM complexes after 3D culturing for 4 or 7 days. The histogram showed the semi quantitative results of fluorescence intensity values of DMP1, CAP and POSTN. Scale bars = 100 μm (**A**), scale bars = 25 μm (**B**). *p < 0.05, **p < 0.01, ***p < 0.001, ****p < 0.0001

ASCs, DFCs, and SHEDs were seeded separately on the surface of pTDM for 4 days. SEM analysis showed a high density of the three cell types covering the surface of pTDM (Fig. 3A). The protein expression was determined by immunofluorescence assay. The results showed that the expression of DMP1 in ASCs/pTDM, and DFCs/pTDM complexes was similar on days 4 and 7, and was higher than that in SHEDs/pTDM complex on day 7. The expression of CAP in ASCs/pTDM complex was significantly higher compared with the expression in the other complexes on day 4. On day 7, the expression of CAP in ASCs/pTDM and DFCs/pTDM complexes was similar, which was higher than that in SHEDs/pTDM complex. POSTN expression in ASCs/pTDM and DFCs/pTDM complexes on day 4 and day 7 were higher compared with the level in SHEDs/pTDM complex, however, there was no significant difference in POSTN expression between ASCs/pTDM and DFCs/pTDM complexes (Fig. 3B).

**Fig. 4:**
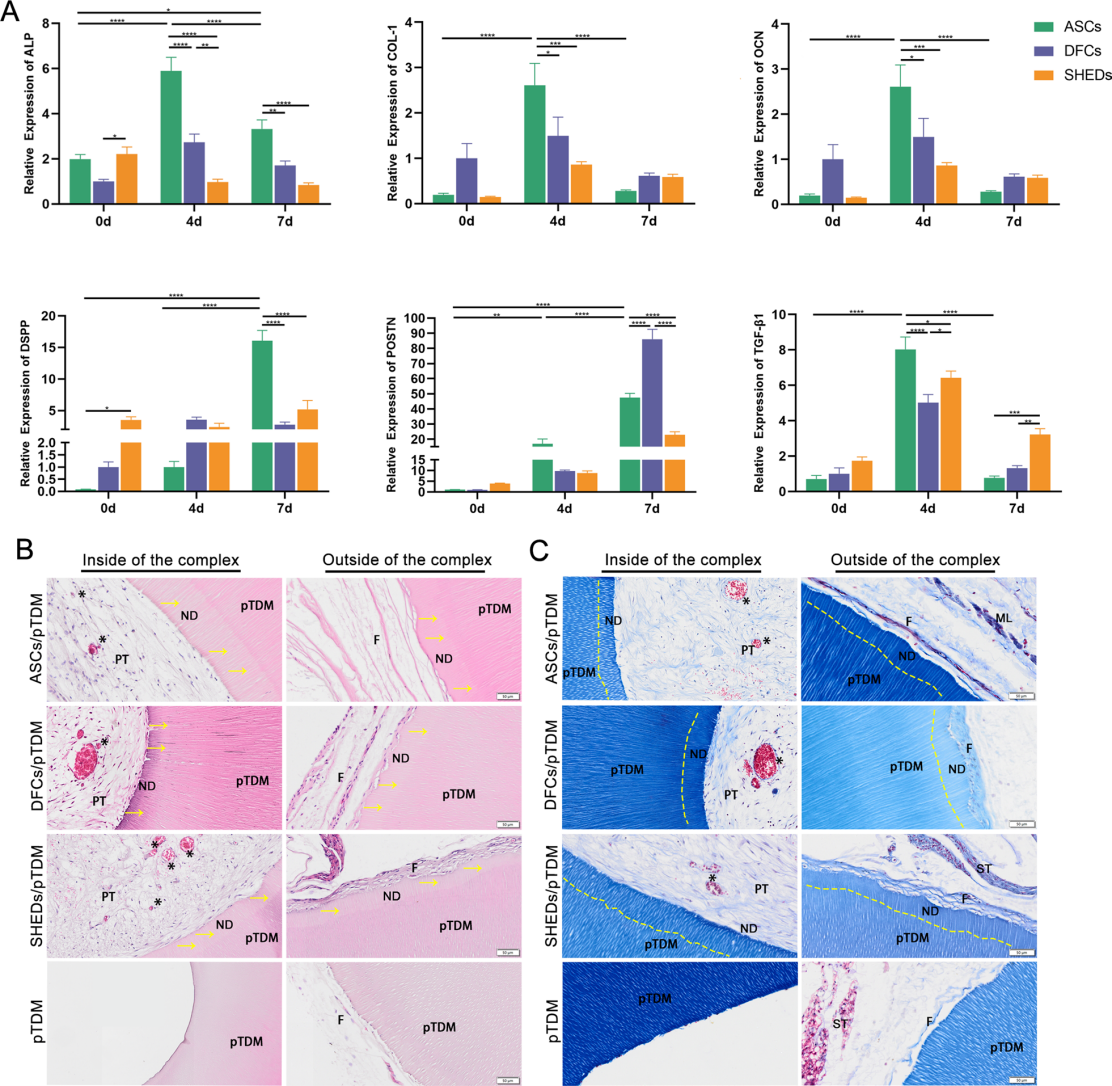
Expression levels of odontogenesis-related genes in three kinds of cells. H&E and Masson’s Trichrome staining results of subcutaneous transplantation in nude mice for 8 weeks. (**A**) Expression levels of odontogenic genes in ASCs/pTDM, DFCs/pTDM, and SHEDs/pTDM complexes after 0, 4, and 7 days of pTDM induction and culture in 3D conditions were determined by RT-qPCR. (**B**) H&E staining and (**C**) Masson’s Trichrome staining showed that pulp-like tissue formation, new dentin formation, and periodontal fiber-like structure formation were observed in ASCs/pTDM, DFCs/pTDM, and SHEDs/pTDM complexes. In pTDM group, only a small amount of periodontal fiber-like structure was observed on the outside. F: periodontal ligament-like fibers, ND: new dentin, ST: skin tissue; PT: pulp tissue, pTDM: treated dentin matrix, *: blood vessel, yellow arrows indicate newly formed dentin. Scale bars = 50 μm. *p < 0.05, ** *p* < 0.01, ***p < 0.001, ****p < 0.0001

After co-culture of the cells with pTDM for 7 days, the mRNA expression levels were determined by RT-qPCR. The expression of *ALP* in ASCs/pTDM complex was significantly higher on day 4 and day 7 compared with the level on day 0 and was significantly higher compared with the level in DFCs/pTDM and SHEDs/pTDM complexes on day 4 and day 7 (Fig. 4A). In addition, *COL-1*, *OCN*, and *TGF-β1* of ASCs/pTDM complex were significantly upregulated on day 4 and slightly upregulated on day 7 with no statistical difference (Fig. 4A). In ASCs/pTDM complex, *COL-1*, *OCN,* and *TGF-β1* were expressed significantly higher levels compared with the expression in DFCs/pTDM and SHEDs/pTDM complexes on day 4. Notably, ASCs/pTDM complex showed a similar expression of *TGF-β1* to that of the DFCs/pTDM complex on day 7, and these complexes displayed significantly lower expression compared with the level in SHEDs/pTDM complex (Fig. 4A). The expression of *DSPP* in ASCs/pTDM complex was significantly higher on day 7 compared with the level on day 0. *DSPP* expression of ASCs/pTDM complex on day 4 was higher compared with the expression level on day 0, however, the difference was not significant (Fig. 4A). ASCs/pTDM complex expressed significantly higher *DSPP* mRNA level on day 7 compared with the expression in DFCs/pTDM and SHEDs/pTDM complexes (Fig. 4A). *POSTN* expression in ASCs/pTDM complex increased gradually from day 0 to day 7, however, the difference was not significant compared with DFCs/pTDM and SHEDs/pTDM complexes on day 0 and day 4 (Fig. 4A). There was no significant difference in *POSTN* expression between DFCs/pTDM and SHEDs/pTDM complexes on day 0 and day 4. However, the expression of *POSTN* in ASCs/pTDM complex was significantly lower compared with the level in DFCs/pTDM complex and significantly higher compared with the expression in SHEDs/pTDM complex on day 7 (Fig. 4A).

### ASCs, DFCs, and SHEDs combined with pTDM induced regeneration of tooth root tissues

Samples were harvested subcutaneously from nude mice after 8 weeks of transplantation. The results of H&E staining (Fig. 4B) and Masson’s Trichrome staining (Fig. 4C) showed that ASCs/pTDM, DFCs/pTDM, and SHEDs/pTDM complexes formed new dentin-like and pulp-like tissues on the insides. Meanwhile, a large number of blood vessels were observed among the newly formed pulp-like tissues in three cell/pTDM groups. However, no new tissue was observed in the medullary cavity of pTDM group. In addition, the analysis showed dentin-like structures were formed on the outsides of the cell/pTDM complexes (ASCs/pTDM, DFCs/pTDM, and SHEDs/pTDM), and a layer of the periodontal fiber-like structure was formed on the outer side next to the newly formed dentin-like structure in cell/pTDM complexes. The findings showed that only a small amount of periodontal fiber-like structure was formed on the outside of pTDM scaffold (blank control group).

**Fig. 5:**
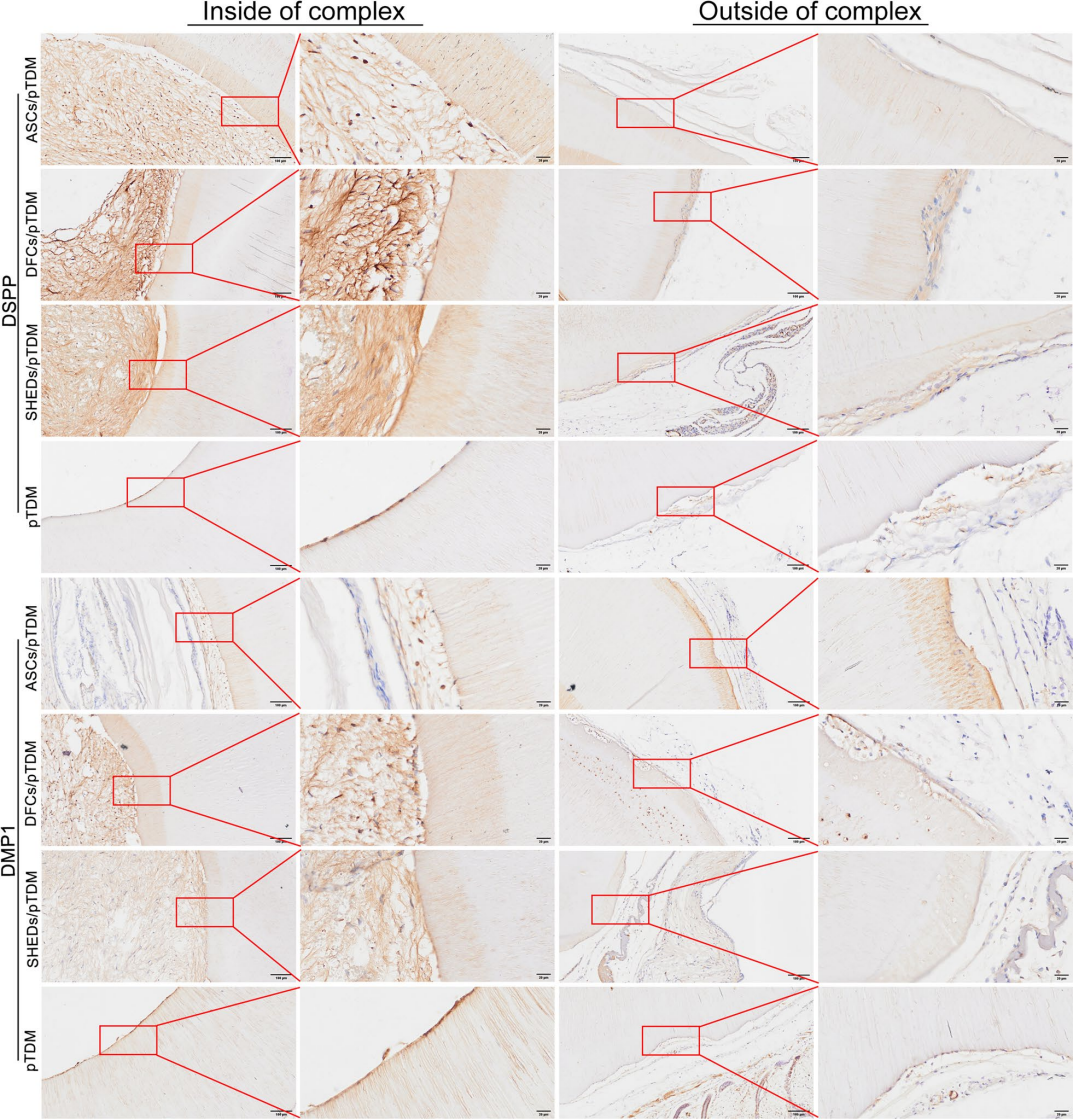
Odontogenic differentiation of bio-root composites was evaluated by immunohistochemistry after 8 weeks of transplantation in vivo. Positive staining indicated DSPP and DMP1 expression in ASCs/pTDM, DFCs/pTDM, and SHEDs/pTDM complexes. Scale bars = 100 μm (the first and third columns), scale bars = 20 μm (the second and fourth columns)

**Fig. 6:**
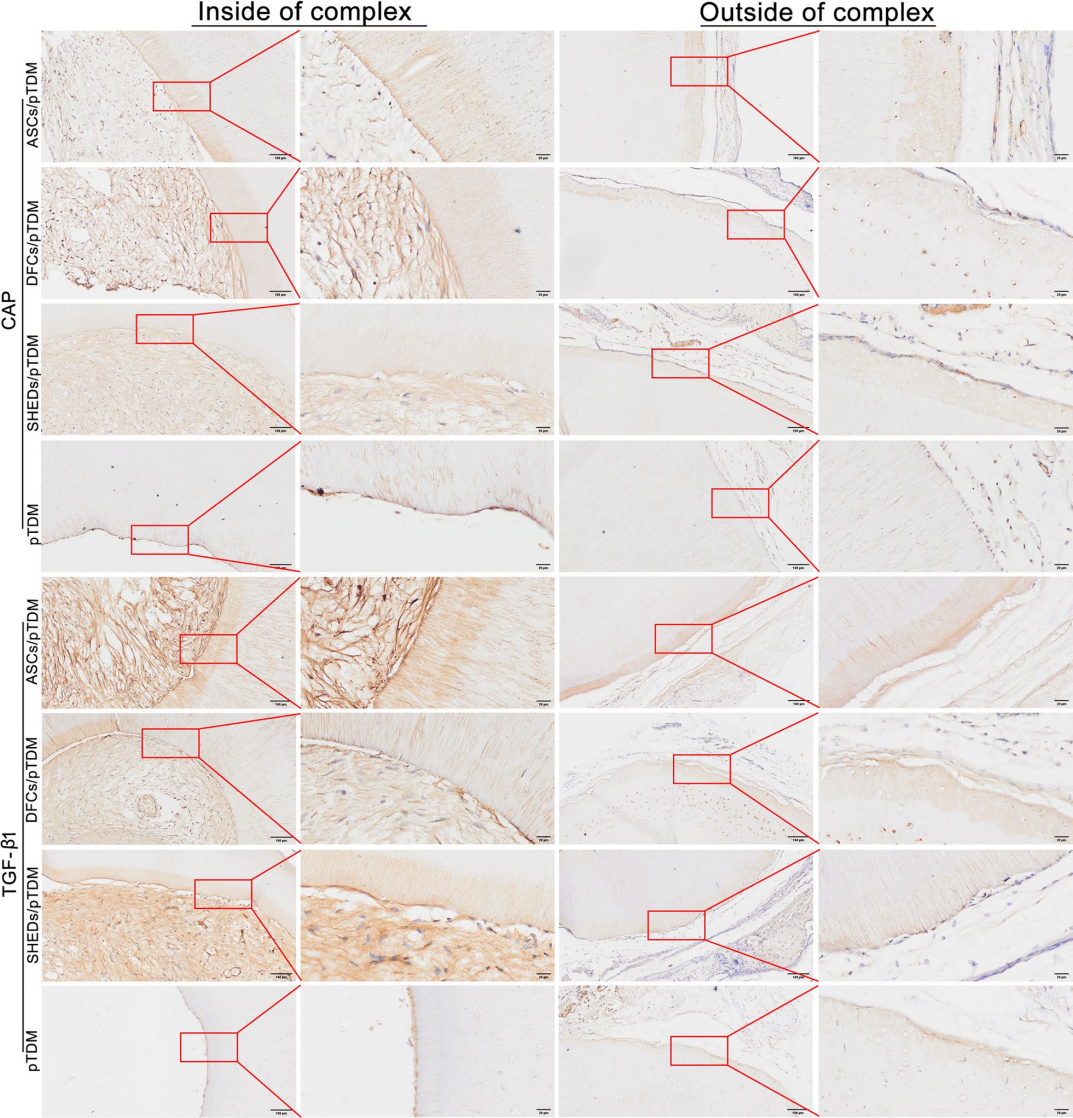
Odontogenic differentiation of bio-root composites was evaluated by immunohistochemistry after 8 weeks of transplantation in vivo. CAP and TGF-β1 expression were indicated by positive staining in ASCs/pTDM, DFCs/pTDM, and SHEDs/pTDM complexes. Scale bars = 100 μm (the first and third columns), scale bars = 20 μm (the second and fourth columns)

Immunohistochemistry analysis demonstrated that dentin-specific proteins DSPP and DMP-1 were expressed in ASCs/pTDM, DFCs/pTDM, and SHEDs/pTDM complexes. In pTDM group, DSPP positive staining was observed on the outside (Fig. 5). In addition, cementum-specific protein, CAP was expressed in the three cell/pTDM complexes groups. Analysis revealed that TGF-β1 which is a fibrous connective tissue marker was expressed in the regenerated tissues of ASCs/pTDM, DFCs/pTDM, and SHEDs/pTDM complexes. Positive staining of CAP and TGF-β1 was observed on the outside of pTDM group (Fig. 6). Moreover, positive expression of the human mitochondrial protein was observed in three groups of the cell/pTDM complexes, but not in pTDM group (Additional file 3: Figure S2).

### Gene expression profile in ASCs/pTDM complex

**Fig. 7:**
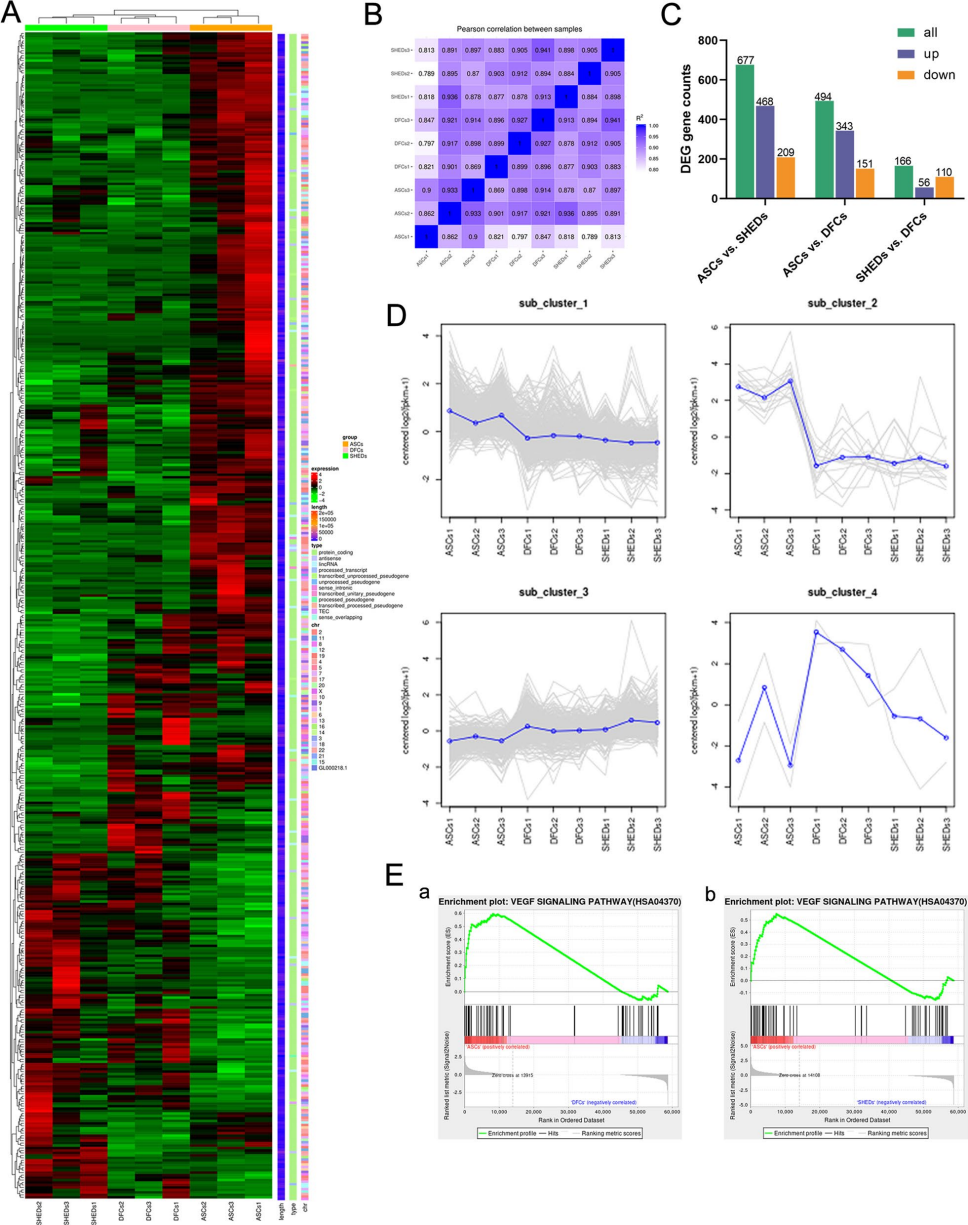
RNA sequencing (RNA-seq) results. (**A**) Heat map of differentially expressed genes in ASCs, DFCs, and SHEDs after induction with pTDM under 3D conditions for 4 days in vitro. Significantly differentially expressed genes in these populations were determined based on RNA-seq results. Cut off: |log2 (foldchange) |> = 1 & Padj <  = 0.05. ASCs1, ASCs2, ASCs3, DFCs1, DFCs2, DFCs3, SHEDs1, SHEDs2, and SHEDs3 represent different biological replicates. (**B**) Pearson correlation analysis of biological samples, a correlation coefficient (R^2^) close to 1 indicates a higher similarity of expression patterns between samples. (**C**) Statistical histogram showed the number of differential genes in different combinations. Note: the number on the column indicated the number of differential genes. (**D**) The log2 (fpkm + 1) was used to generate a plot after centralized correction to obtain a gene list corresponding to each subset of the clustering line graph based on expression levels of differentially expressed genes in figure **A**. The abscissa indicated the sample name, and the ordinate indicated the expression value. The logarithm-centered corrected value was shown. The gray line in each subgraph represented the relative corrected gene expression levels of genes in a cluster among different experimental groups. The blue line represented the average relative corrected gene expression level of all genes in the cluster among different experimental groups. (**E**) Gene set enrichment analysis (GSEA) based on RNA-Seq data of ASCs/pTDM complex

**Fig. 8:**
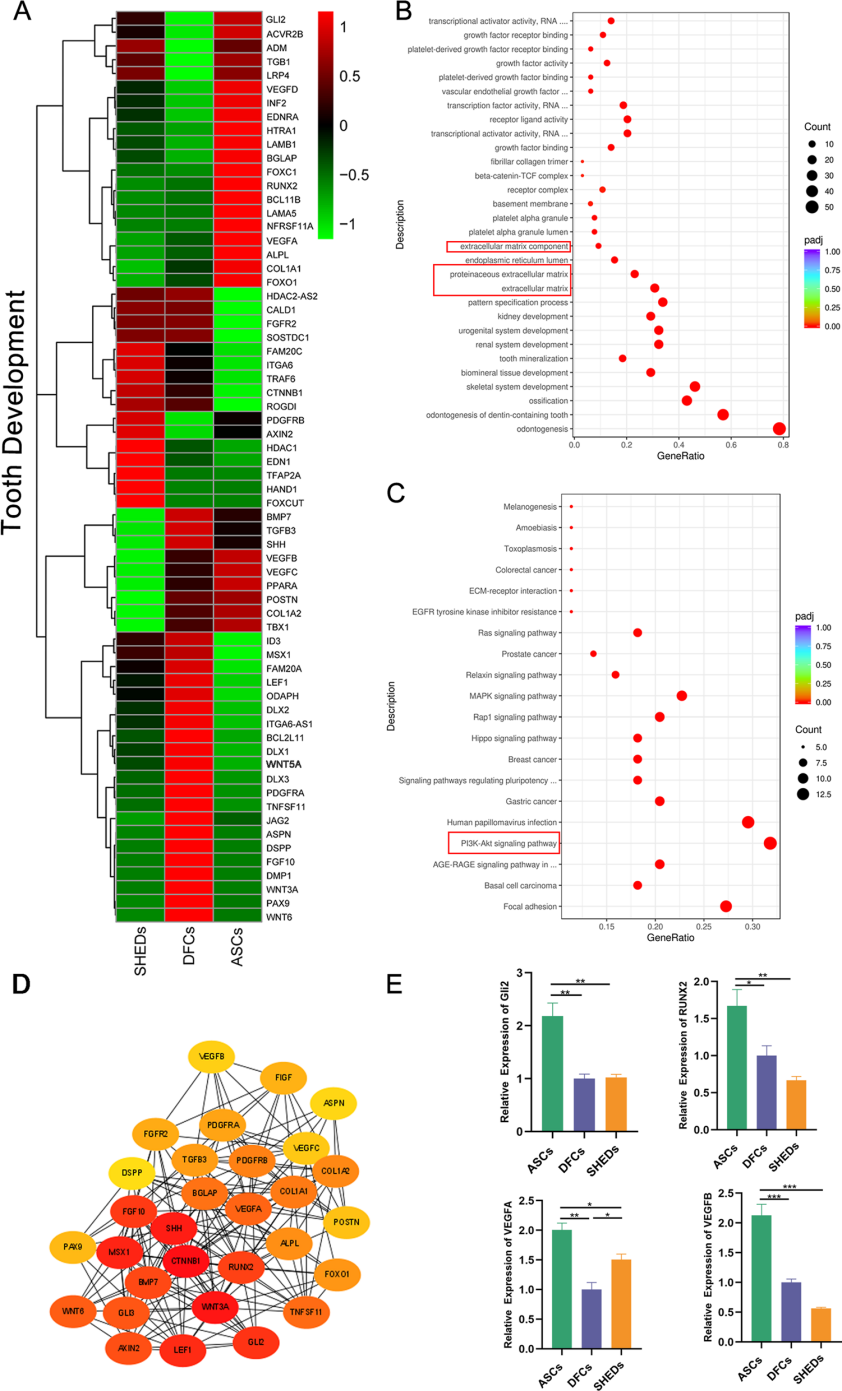
Expression levels of tooth development-related genes in cell/pTDM complexes. (**A**) Heat map of tooth development-related genes based on RNA-seq analysis of ASCs, DFCs, and SHEDs after induction with pTDM under 3D conditions for 4 days in vitro. Cut off: |log2 (foldchange) |> = 1 & Padj <  = 0.05. (**B**) GO analysis showed that the tooth development-related genes were enriched in odontogenesis, calcification, and bone development. The red box indicated extracellular matrix-related processes. (**C**) KEGG analysis showed that the top significantly enriched signaling pathway was the PI3K-Akt signal pathway, marked with a red box. (**D**) PPI analysis of these tooth development-related proteins. (**E**) The mRNA expression levels of *Gli2*, *RUNX2*, *VEGFA*, and *VEGFB* were validated by RT-qPCR. *p < 0.05, **p < 0.01, ***p < 0.001

Transcriptome analysis was performed to explore potential molecular mechanisms of the functions of ASCs/pTDM, DFCs/pTDM, and SHEDs/pTDM complexes in odontogenesis. Pearson correlation analysis showed that the repeatability among the three groups of cell biological samples was good (Fig. 7B). Differentially expressed genes were identified in these cell/pTDM complexes and presented as a heat map (Fig. 7A). Analysis showed a total of 677 differential genes in ASCs vs. SHEDs group, with 468 upregulated genes, and 209 downregulated genes (Fig. 7C). The total number of differentially expressed genes in ASCs vs. DFCs group was 494, with 343 upregulated genes and 151 downregulated genes (Fig. 7C). Analysis of DFCs vs. SHEDs group revealed a total of 166 differentially expressed genes, and out of these genes 56 were upregulated and 110 were downregulated (Fig. 7C). Differential expressed genes were mainly clustered into four groups (Fig. 7D). The sub_cluster _2 group comprised genes highly expressed in ASCs/pTDM complex. Gene set enrichment analysis (GSEA) based on RNA-Seq results showed that ASCs/pTDM complex was significantly enriched in VEGF singling pathway compared with enrichment of DFCs/pTDM and SHEDs/pTDM complexes (Fig. 7E). GO analysis demonstrated that genes related to extracellular matrix (ECM) synthesis were significantly enriched in ASCs/pTDM complex (Additional file 4: Figure S3A). Moreover, KEGG signal pathway analysis showed that significantly enriched pathways in ASCs/pTDM complex mainly included PI3K-Akt signaling pathway, focal adhesion, and the cytokine-cytokine receptor interaction pathway (Additional file 4: Figure S3B). Notably, the PI3K-Akt signaling pathway was the most enriched. The findings showed that the three highly expressed genes in ASCs/pTDM complex were *KDR*, *COMP*, and *FGF18*, as shown by RT-qPCR analysis, and were implicated in the PI3K-Akt signaling pathway (Additional file 4: Figure S3C). These findings were consistent with the RNA-seq results. Analysis of genes related to ECM revealed that the two genes highly expressed in ASCs/pTDM complex were *COL16A1* and *MMP8*. RT-qPCR results were consistent with the sequencing results (Additional file 4: Figure S3C). Cluster analysis results showed that tooth development-related genes were highly expressed in the three groups of complexes (Fig. 8A). Osteogenic, ECM-related, and angiogenesis-related genes such as *ALP*, *OCN*, *COL-1*, *VEGFA*, and *VEGFB* were highly expressed in ASCs/pTDM complex (Fig. 8A). Moreover, the expressions of *ALP*, *COL-1*, *OCN*, *TGF-β1*, *DSPP*, and *POSTN* in ASCs/pTDM, DFCs/pTDM, and SHEDs/pTDM complexes were consistent with the RT-qPCR findings (Fig. 4A). GO analysis showed that the screened genes were significantly enriched in dentin formation and calcification processes. Meanwhile, ECM-related biological processes were included (Fig. 8B). KEGG pathway analysis showed that these tooth development-related genes were significantly enriched in the PI3K-Akt pathway (Fig. 8C). PPI analysis was performed to explore the functional interactions between these tooth development-related proteins. Analysis showed that the top ten proteins that played a central role in the network were CTNNB1, Wnt3a, Shh, Msx1, LEF1, Gli2, FGF10, RUNX2, BMP7, and AXIN2 (Fig. 8D). In addition, the expression of *RUNX2*, *Gli2*, *VEGFA*, and *VEGFB*, which was significantly highly expressed in ASCs/pTDM complex and implicated in the tooth development process (Fig. 8A) was validated by RT-qPCR analysis. The findings from RT-qPCR analysis were consistent with the RNA-seq results (Fig. 8E).

## Discussion

Stem cells based bio-root regeneration is a promising treatment approach for tooth loss [39]. Dental-derived stem cells such as DFCs, SHEDs, and DPCs have been used as seed cells for bio-root regeneration in previous studies [10, 11, 13, 14, 24, 25]. However, it is challenging to obtain a large supply of these cells [14, 26, 27]. Therefore, it is necessary to explore new seed cells which are available in higher amounts.

ASCs are safe and have higher practical applications owing to their high availability from liposuction, low immunity, and low incidence rate. The use of autologous adipose tissue involves less ethical controversy [40–42]. The findings of the present study showed no significant differences in the expression of immunophenotype markers (CD14, CD19, CD45, CD73, CD90, CD105, and CD146) between ASCs and dental-derived stem cells, which implies that ASCs meet the requirements of mesenchymal stem cells [43, 44]. In the current study, ASCs exhibited excellent osteogenesis and neurogenesis activities similar to dental-derived stem cells. Previous studies report that ASCs present high osteogenesis activity [45]. Moreover, scaffolds conducive to osteogenesis inoculated with ASCs can undergo osteogenic differentiation without stimulation in vivo [46]. ASCs have been proved to promote neurite growth in vitro [47] and enhance peripheral nerve regeneration in vivo [48]. These are consistent with the results of the current study. In addition, regenerative characteristics of ASCs include secretion of restorative growth factors [49, 50], promoting angiogenesis and wound healing, and potentially promoting growth or development of new tissues [50, 51]. The findings of the current study showed that ASCs expressed pluripotent stem cell markers including *Nanog* and *Sox2* in P1-P7, and the expression levels were similar to that of these markers in dental-derived stem cells, especially SHEDs. Previous studies have proved that *Nanog* delays aging of hair follicle-derived mesenchymal stem cells by up-regulating the expression of *Pbx1* and activating the Akt signaling pathway [52]. Notably, short-term cyclic expression of several genes including *Sox2* improves cellular senescence characteristics of aging mouse model [30]. These findings may explain why the staining of senescent cells was not evident even when cultured to P13 in vitro, implying that ASCs have a similarly excellent stem cell pluripotency to that of dental-derived stem cells. The current study showed that ASCs expressed mesoderm marker (*PDGFRα*) and ectoderm marker (*β-III tubulin*), which is consistent with the previous research results, that is, ASCs can transform to ectoderm, mesoderm, and endoderm cells [53]. However, dental-derived stem cells originated from the cranial neural crest, are ectoderm cells [54, 55], suggesting that ASCs may differentiate into dental-lineage cells. These findings demonstrate the high application potential of ASCs in bio-root regeneration. Further analyses were conducted to explore the potential application of ASCs in bio-root regeneration.

The regional microenvironment is an indispensable factor in maintaining normal cell growth, proliferation, differentiation, and metabolism [56]. In earlier studies, researchers cultured cells into cell sheets and combined them with TDM to construct bio-root complexes [11, 14]. This method cannot ensure the tight adhesion of cell sheets to TDM and the homogeneous distribution of cells on the surface of TDM. The current research results show that the 3D culture system constructed in this study can solve the problems of cell adhesion density and tightness, and promote cell proliferation and differentiation. For one thing, the protein composition of TDM contains cell adhesion-related proteins, such as fibronectin 1, vitronectin, and laminin, to ensure the tight adhesion of cells attached to its surface [8]. Ultra-low adhesion dishes allow more cells to grow on the surface of pTDM rather than traditional dishes, resulting in most cells adhering to the bottom of the dishes. For another thing, the 3D odontogenic microenvironment provided by pTDM may induce cell differentiation on its surface by both direct and indirect contact rather than releasing soluble factors to the culture media. This 3D culture system shows high potential in dental tissue engineering regeneration owing to the high cell inoculation rate and good operability. Altogether, the 3D culture system provides a platform to mimic the microenvironment of stem cells differentiation in vivo.

In a variety of previous studies, when it comes to the detection of odontogenic differentiation ability of cells in vitro, an odontogenic induction medium was often used, such as ascorbic acid, β-glycerophosphate, and monopotassium phosphate were added [14, 57]. Due to the natural superiority of the scaffold material in this study, the co-culture of cells and pTDM in a 3D culture system was used instead of the traditional application of induction medium when studying the odontogenic differentiation ability of cells in vitro. DMP1 and CAP are specific proteins in dentin, cementum [58, 59], which play an important role in the mineralization of key components of the root, including dentin and cementum [59]. Whereas POSTN is a protein located in the periodontal ligament and plays an important role in the development and eruption of teeth. Moreover, POSTN protein is implicated in the integrity and function of the periodontal ligament [60]. The findings of the present study showed that the protein expression levels of these markers were significantly high in ASCs/pTDM and DFCs/pTDM complexes, indicating induction of pTDM enables ASCs to have odontogenic differentiation capacity similar to dental-derived stem cells. The gene expression findings demonstrate that ASCs/pTDM complex has higher osteogenic activity compared with DFCs/pTDM and SHEDs/pTDM complexes as indicated by upregulation of *ALP*, *COL-1*, and *OCN* expression, which are markers of osteogenic differentiation. These markers play important roles in the formation of calcium phosphate minerals in dentin and cementum [61–63]. *DSPP* is the terminal phenotypic marker of mature odontoblasts and plays an important role in dentin formation [64]. The findings displayed a significantly high mRNA expression level of *DSPP* in ASCs/pTDM complex indicating ASCs cultured in the natural odontogenic microenvironment provided by pTDM present high odontogenic differentiation potential. *TGF-β1* is a marker for the maintenance and regeneration of periodontal ligament tissue [65]. Furthermore, *TGF-β1* is one of the most abundant cytokines in the bone matrix and is implicated in the regulation of proliferation and differentiation of osteoblasts [66, 67]. *TGF-β1* mRNA expression level was significantly high in ASCs/pTDM complex according to the results of the present study, implying the high potential of ASCs/pTDM in periodontal tissue regeneration. In addition, analysis of ASCs/pTDM, DFCs/pTDM, and SHEDs/pTDM complexes showed that most of the changes in the expression of osteogenesis-related genes detected were opposite to the changes of genes related to odontogenesis. The results displayed the expression of osteogenesis-related genes such as *COL-1*, *OCN*, and *TGF-β1* was the highest expression on day 4 and the expression decreased on day 7 of culture. Whereas the expression of odontogenesis-related genes such as *DSPP* and *POSTN* showed a gradual increase with the highest expression observed on day 7. This finding can be attributed to differences in molecular mechanisms involved in the regulation of osteogenesis and odontogenesis processes. In summary, pTDM, as a bioactive scaffold material, can induce the odontogenic differentiation of non-dental-derived cells (ASCs), providing the possibility for ASCs to become bio-root regeneration seed cells.

Findings from previous studies revealed that the application of dental-derived stem cells alone in vivo results in insufficient dentin tissue formation [68, 69]. In this study, ASCs, DFCs, and SHEDs combined with pTDM were subcutaneously transplanted into nude mice to further explore the odontogenic differentiation potential of cells in vivo. Histomorphological analysis showed that ASCs/pTDM and dental-derived stem cells/pTDM complexes generated continuous dentin-like tissue, pulp-like tissue, periodontal fiber-like tissue on the surfaces of pTDM, which may be attributed to the pTDM induced ASCs have the similar odontogenic differentiation potential as dental-derived stem cells on the one hand, and on the other hand, it is attributed to the root morphology provided by pTDM, which provides attachment for regenerated tissues. In addition, all regenerated tissues in the cell/pTDM complexes expressed dentin proteins (DMP1 and DSPP), cement protein (CAP), and a marker of fibrous connective tissue (TGF-β1). This further confirmed that ASCs/pTDM complex has a similar capacity for regeneration of dentin-like tissue and periodontal ligament-like fibrous tissue as dental-derived stem cells. Furthermore, positive expression of anti-human mitochondrial antibodies in the three groups reveals that cells in cell/pTDM complexes play a predominant role in odontogenic differentiation. These results suggested that pTDM can induce odontogenic differentiation of ASCs in vivo.

Bioinformatics analysis showed differential gene expression between dental-derived stem cells/pTDM and ASCs/pTDM complexes. The finding from GO analysis showed that the highly expressed genes in ASCs/pTDM complex were significantly enriched in ECM-related biological processes. ECM proteins are implicated in the regulation of cell adhesion, proliferation, angiogenesis, or apoptosis [70]. *COL16A1* is a common extracellular matrix protein [71]. ECM exists in all tissues and is a highly dynamic structure that undergoes controlled remodeling. Matrix metalloproteinases (including *MMP8*) play key roles in the remodeling process [72]. The high expression levels of *COL16A1* and *MMP8,* and GO enriched terms in tooth development genes set in ASCs/pTDM complex observed in the present study indicate that the formation of ECM presents a dynamic structure and is constantly transformed to control tissue balance during the bio-root regeneration process of ASCs/pTDM complex. KEGG analysis and GSEA enrichment analysis showed that the significantly enriched pathways were PI3K-Akt and VEGF signaling pathways. *VEGFR-2*, also known as *KDR* promotes vascular regeneration [73]. Cartilage Oligomeric Matrix Protein (*COMP*) binds smad protein and upregulates *BMP-2* which induces intracellular signal, ultimately increasing the expression of *BMP* receptor thus regulating bone regeneration [74]. Fibroblast growth factor-18 (*FGF18*) induces bone differentiation on rat BMSC (rBMSCs) [75]. These genes involved in the PI3K-Akt signaling pathway were highly expressed in ASCs/pTDM complex, indicating that the PI3K-Akt pathway is probably involved in angiogenesis and osteogenesis. Furthermore, VEGF plays an important role in angiogenesis [76]. *VEGFA* is a powerful stimulator of angiogenesis [77] and *VEGFB* is a homolog of VEGF which is implicated in the survival of blood vessels (78). *VEGFA* and *VEGFB* were highly expressed in the ASCs/pTDM complex in the present study, implying that the two factors may positively regulate the formation of dental pulp-like tissue in animal experiments. In brief, these results suggest that ASCs/pTDM complex may present more advantages in revascularization of bio-root, such as pulp tissue regeneration, periodontal vascular regeneration, etc., but it needs to be further explored.

In the current study, the biological characteristics of dental-derived stem cells and ASCs were compared in vitro. Moreover, the odontogenic differentiation potential of ASCs under 3D culture was verified in vitro, and in vivo. The findings demonstrate that ASCs can be used as seed cells for bio-root regeneration. However, the present study had some limitations. For instance, the potential molecular mechanism of dental-derived stem cells and ASCs underlying peak expression levels of osteogenic and odontogenic markers at different times should be explored. Moreover, the molecular mechanism underlying the differences in gene expression between dental-derived stem cells and ASCs should be explored. Furthermore, the animal experiment studies were only conducted with subcutaneous transplantation in nude mice, which is different from the microenvironment of alveolar bone in situ. Thus, further orthotopic transplantation experiments in large animals should be conducted.

## Conclusion

The current findings suggest that pTDM is a scaffold material with strong odontogenic inducibility, and the induced ASCs have similar potential to dental-derived stem cells in bio-root regeneration. ASCs exhibited good cell biological characteristics and odontogenic differentiation ability in vitro. Moreover, 3D cultured ASCs/pTDM complex promoted regeneration of dentin-like, pulp-like, and periodontal fibrous-like tissues in vivo. Analysis indicates that the PI3K-Akt pathway, VEGF signaling pathway, and ECM-related genes play significant roles in bio-root regeneration process induced by the ASCs/pTDM complex. In summary, ASCs are potential seed cell candidates for bio-root regeneration, as they show high effectiveness in bio-root regeneration. Abundant sources of pTDM and ASCs laid a basis for further experimental research and future clinical application of bio-root regeneration.

## Supplementary Information

**Additional file 1: Table S1**. The RT-qPCR primer sequences adopted in the current study.

**Additional file 2.** **Fig. S1**. Biological characteristics of ASCs, DFCs, and SHEDs. (A a, b, and c) Cell cycle results of the three cell types. (A d, e, and f) Quantitative analysis indicated that there was no difference in the DNA content of the three cell types in the G1 phase, G2 phase, and S phase. (B a, b and c) Apoptosis rates of the three cell types. (B d) Quantitative analysis showed that the difference in the apoptosis rates was not statistically significant. (C) The cell proliferation rate of DFCs was higher compared with that of ASCs and SHEDs, and the cell proliferation rate of ASCs was higher compared with that of SHEDs as shown by CCK-8 assay. (D) β-galactosidase staining (positive cells performed dark blue) showed that the three cell types did not exhibit senescent cells. Scale bars = 500 μm. **p < 0.01, ***p < 0.001, ****p < 0.0001.

**Additiona1 file 3.** **Fig. S2**. Odontogenic differentiation of bio-root composites was evaluated by immunohistochemistry after 8 weeks of transplantation in vivo. Positive staining of Mitochondria was observed in three cell/pTDM groups. PBS was used as the negative control. Scale bars = 100 μm (the first and third columns), scale bars = 20 μm (the second and fourth columns).

**Additional file 4. Fig. S3**. Enriched terms related to the genes in ASCs/pTDM complex. (A) GO analysis showed that upregulated genes in ASCs/pTDM complex were significantly enriched in the extracellular matrix marked with a red box. (B) KEGG analysis showed that genes upregulated in ASCs/pTDM complex were significantly enriched in PI3K-Akt, focal adhesion, and cytokine-cytokine receptor interaction pathways. The PI3K-Akt pathway was marked with a red box. (C) mRNA expression levels of genes implicated in extracellular matrix and PI3K-Akt signaling pathway in cell/pTDM complexes (ASCs/pTDM, DFCs/pTDM, and SHEDs/pTDM) cultured for 4 days in vitro as indicated by RT-qPCR analysis. *p < 0.05, **p < 0.01, ***p < 0.001.

## Data Availability

The RNA-sequencing datasets supporting the conclusions of this article are available in the National Centre for Biotechnology Information Gene Expression Omnibus repository (NCBI GEO, www.ncbi.nlm.nih.gov/geo) [accession number GSE203020].

## Abbreviations

ASCs: Adipose-derived stromal/stem cells
DFCs: Dental follicle cells
SHEDs: Stem cells from human exfoliated deciduous teeth
2D: Two-dimensional
3D: Three-dimensional
RNA-seq: RNA sequencing
TDM: Treated dentin matrix
hTDM: Human treated dentin matrix
pTDM: Porcine treated dentin matrix
ELVs-H1: Exosome-like vesicles derived from Hertwig's epithelial root sheath cells
DPCs: Dental papilla cells
PBS: Phosphate-buffered saline
α-MEM: α-Minimum essential medium
FBS: Fetal bovine serum
SA-β-gal: Senescence-associated β-galactosidase
RT-qPCR: Quantitative real-time PCR
SEM: Scanning electron microscopy
GO: Gene Ontology
