# Supplementary material for: Adipose-derived stromal/stem cells are verified to be potential seed candidates for bio-root regeneration in three-dimensional culture
Source: Stem Cell Res Ther. 2022 Jun 3;13:234. doi: 10.1186/s13287-022-02907-y (PMC9166419; doi:10.1186/s13287-022-02907-y)
Supplement: Supplementary file 1 — Additional file 1: Table S1. The RT-qPCR primer sequences adopted in the current study. [file 13287_2022_2907_MOESM1_ESM.docx]

**Table 1. Oligonucleotide primer sequences**

| **Target cDNA** | **Primer sequence(5'--3')** | **Product length** | **Accession No.** |
| --- | --- | --- | --- |
| Nanog | F GTCCCAAAGGCAAACAACCC | 108 | NM_024865.4 |
|  | R GCTGGGTGGAAGAGAACACA |  |  |
| Sox2 | F CTACAGCATGATGCAGGACCA | 130 | NM_003106.4 |
|  | R CGAGCTGGTCATGGAGTTGTA |  |  |
| β-Ⅲ tubulin | F AGATCGGGGCCAAGTTCTG | 142 | NM_006086.4 |
|  | R CGAGGCACGTACTTGTGAGA |  |  |
| PDGFRα | F TGGCAGTACCCCATGTCTGAA | 88 | NM_001347830.2 |
|  | R CCAAGACCGTCACAAAAAGGC |  |  |
| DSPP | F TTTGGGCAGTAGCATGGGC | 199 | NM_014208.3 |
|  | R CCATCTTGGGTATTCTCTTGCCT |  |  |
| DMP1 | F CACTCAAGATTCAGGTGGCAG | 75 | NM_001079911.3 |
|  | R TCTGAGATGCGAGACTTCCTAAA |  |  |
| POSTN | F CTCATAGTCGTATCAGGGGTCG | 138 | NM_001135935.2 |
|  | R ACACAGTCGTTTTCTGTCCAC |  |  |
| OCN | F CTCACACTCCTCGCCCTATTG | 166 | NM_199173.6 |
|  | R CTCCCAGCCATTGATACAGGTAG |  |  |
| COL-1 | F GAGGGCCAAGACGAAGACATC | 140 | NM_000088.4 |
|  | R CAGATCACGTCATCGCACAAC |  |  |
| RUNX2 | F CTTTACTTACACCCCGCCAGTC | 146 | NM_001015051.4 |
|  | R AGAGATATGGAGTGCTGCTGGTC |  |  |
| TGF-β1 | F ATTTATTGAGCACCTTGGGCAC | 130 | NM_000660.7 |
|  | R TCTCTGGGCTTGTTTCCTCAC |  |  |
| ALP | F TAAGGACATCGCCTACCAGCTC | 170 | NM_001127501.4 |
|  | R TCTTCCAGGTGTCAACGAGGT |  |  |
| KDR | F CGGTCAACAAAGTCGGGAGA | 123 | NM_002253.4 |
|  | R CAGTGCACCACAAAGACACG |  |  |
| COMP | F AAGAACGACGACCAAAAGGAC | 154 | NM_000095.3 |
|  | R CATCCCCTATACCATCGCCA |  |  |
| FGF18 | F GTACGTGGGCTTCACCAAGA | 140 | NM_003862.3 |
|  | R TGGTCACCGTCGTGTACTTG |  |  |
| COL16A1 | F GACTCCGTTGGGAATAAATGGC | 199 | NM_001856.4 |
|  | R TAAACGGGAAAAGGAGGGCA |  |  |
| MMP8 | F AGCTGTCAGAGGCTGAGGTA | 189 | NM_001304442.2 |
|  | R GCTGAAAGGCATGAGCAAGG |  |  |
| Gli2 | F GCATCTCTTGCCACCATTCC | 93 | NM_001374353.1 |
|  | R ACACCGTGGACAGAATGAGG |  |  |
| VEGFA | F TCTCCCTGATCGGTGACAGT | 107 | NM_001025366.3 |
|  | R AAGGAATGTGTGCTGGGGAG |  |  |
| VEGFB | F CACAGCCAGTGTGAATGCAG | 153 | NM_003377.5 |
|  | R AGTGGGATGGGTGATGTCAG |  |  |
| GAPDH | F CTTTGGTATCGTGGAAGGACTC | 132 | NM_001357943.2 |
|  | R GTAGAGGCAGGGATGATGTTCT |  |  |
