# Supplementary figures and images for: Adipose-derived stromal/stem cells are verified to be potential seed candidates for bio-root regeneration in three-dimensional culture
Source: Stem Cell Res Ther. 2022 Jun 3;13:234. doi: 10.1186/s13287-022-02907-y (PMC9166419; doi:10.1186/s13287-022-02907-y)

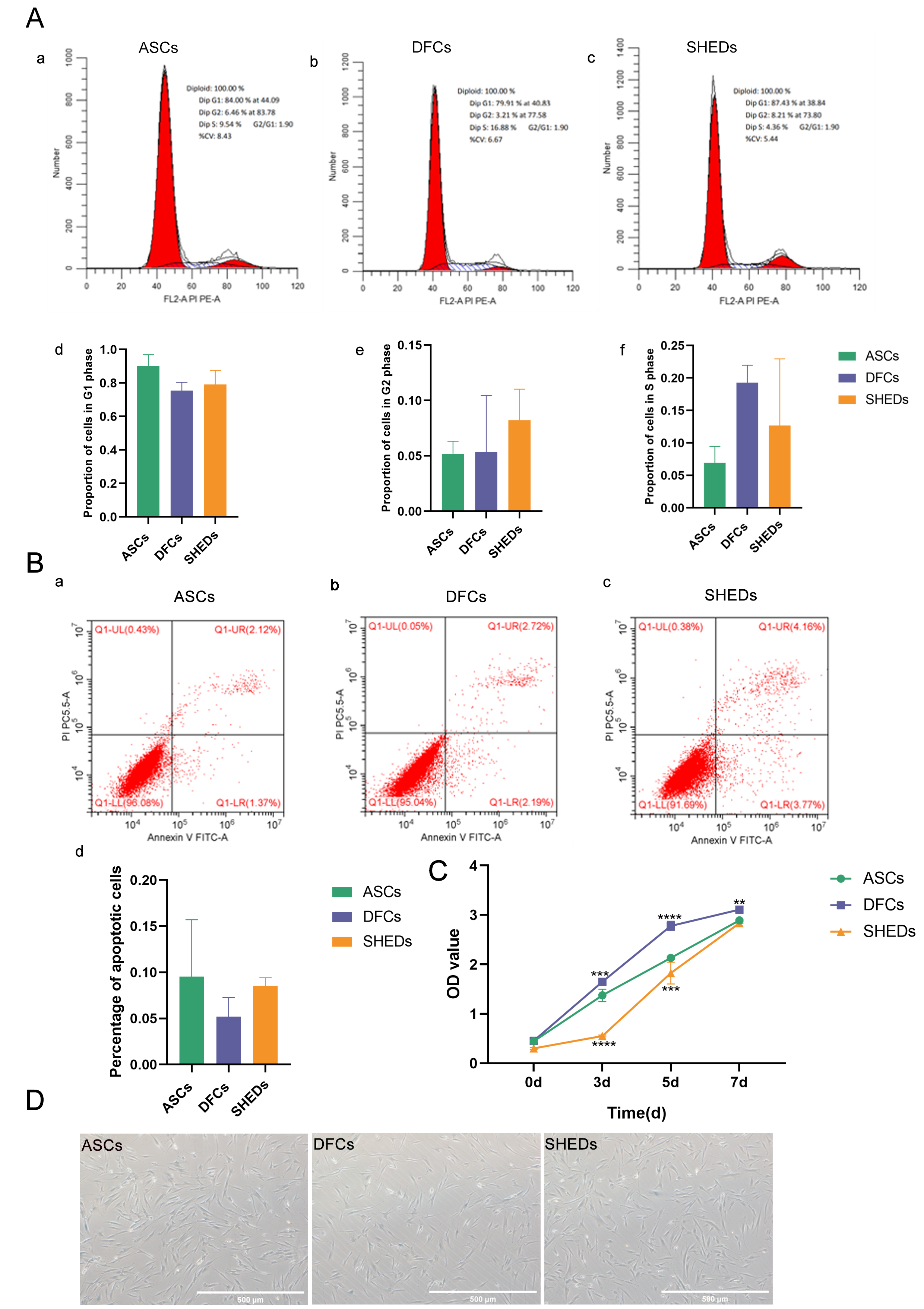

Supplement: Supplementary file 2 — Additional file 2. Fig. S1. Biological characteristics of ASCs, DFCs, and SHEDs. (A a, b, and c) Cell cycle results of the three cell types. (A d, e, and f) Quantitative analysis indicated that there was no difference in the DNA content of the three cell types in the G1 phase, G2 phase, and S phase. (B a, b and c) Apoptosis rates of the three cell types. (B d) Quantitative analysis showed that the difference in the apoptosis rates was not statistically significant. (C) The cell proliferation rate of DFCs was higher compared with that of ASCs and SHEDs, and the cell proliferation rate of ASCs was higher compared with that of SHEDs as shown by CCK-8 assay. (D) β-galactosidase staining (positive cells performed dark blue) showed that the three cell types did not exhibit senescent cells. Scale bars = 500 μm. **p < 0.01, ***p < 0.001, ****p < 0.0001. [file 13287_2022_2907_MOESM2_ESM.tif]

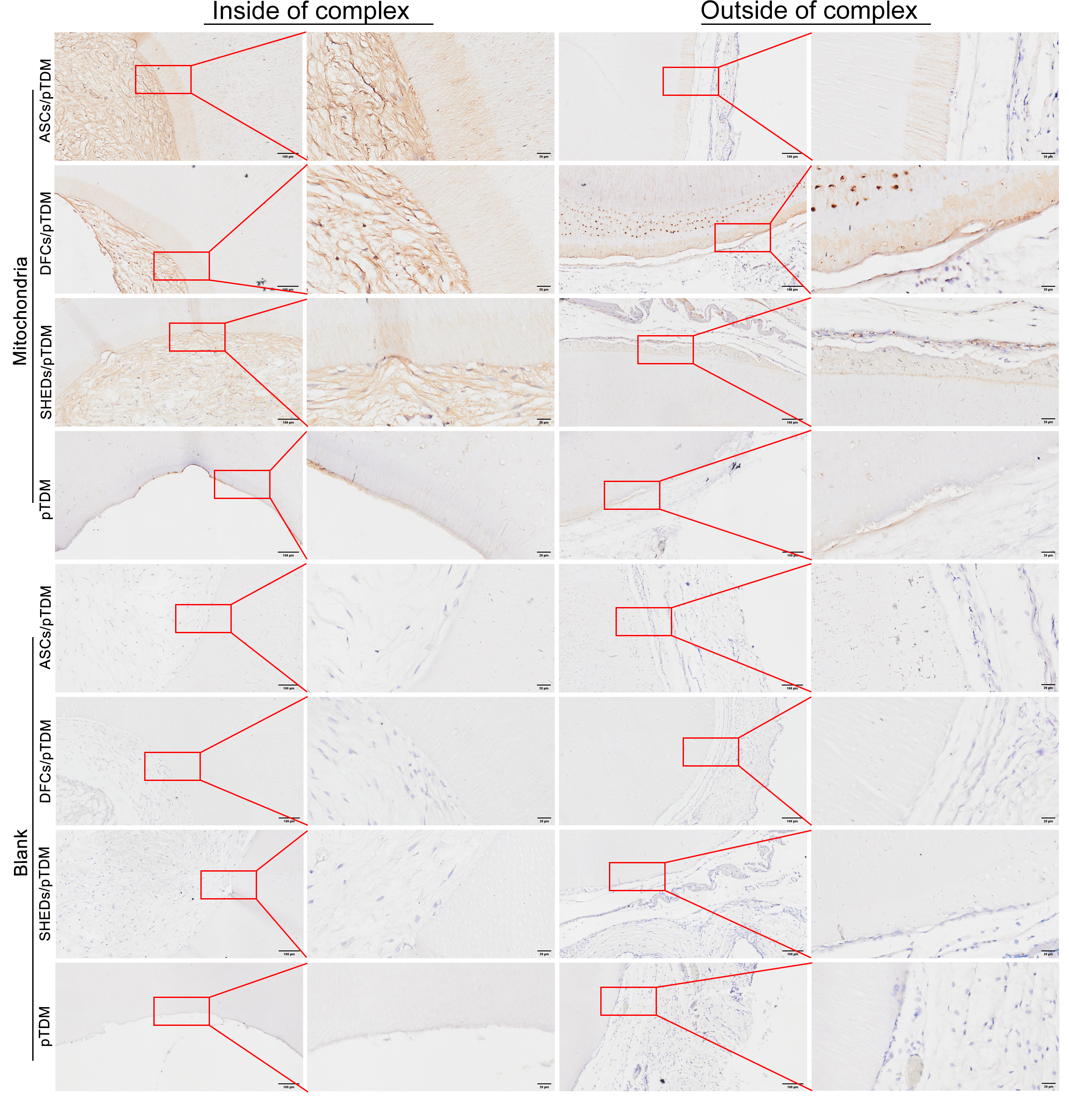

Supplement: Supplementary file 3 — Additiona1 file 3. Fig. S2. Odontogenic differentiation of bio-root composites was evaluated by immunohistochemistry after 8 weeks of transplantation in vivo. Positive staining of Mitochondria was observed in three cell/pTDM groups. PBS was used as the negative control. Scale bars = 100 μm (the first and third columns), scale bars = 20 μm (the second and fourth columns). [file 13287_2022_2907_MOESM3_ESM.tif]

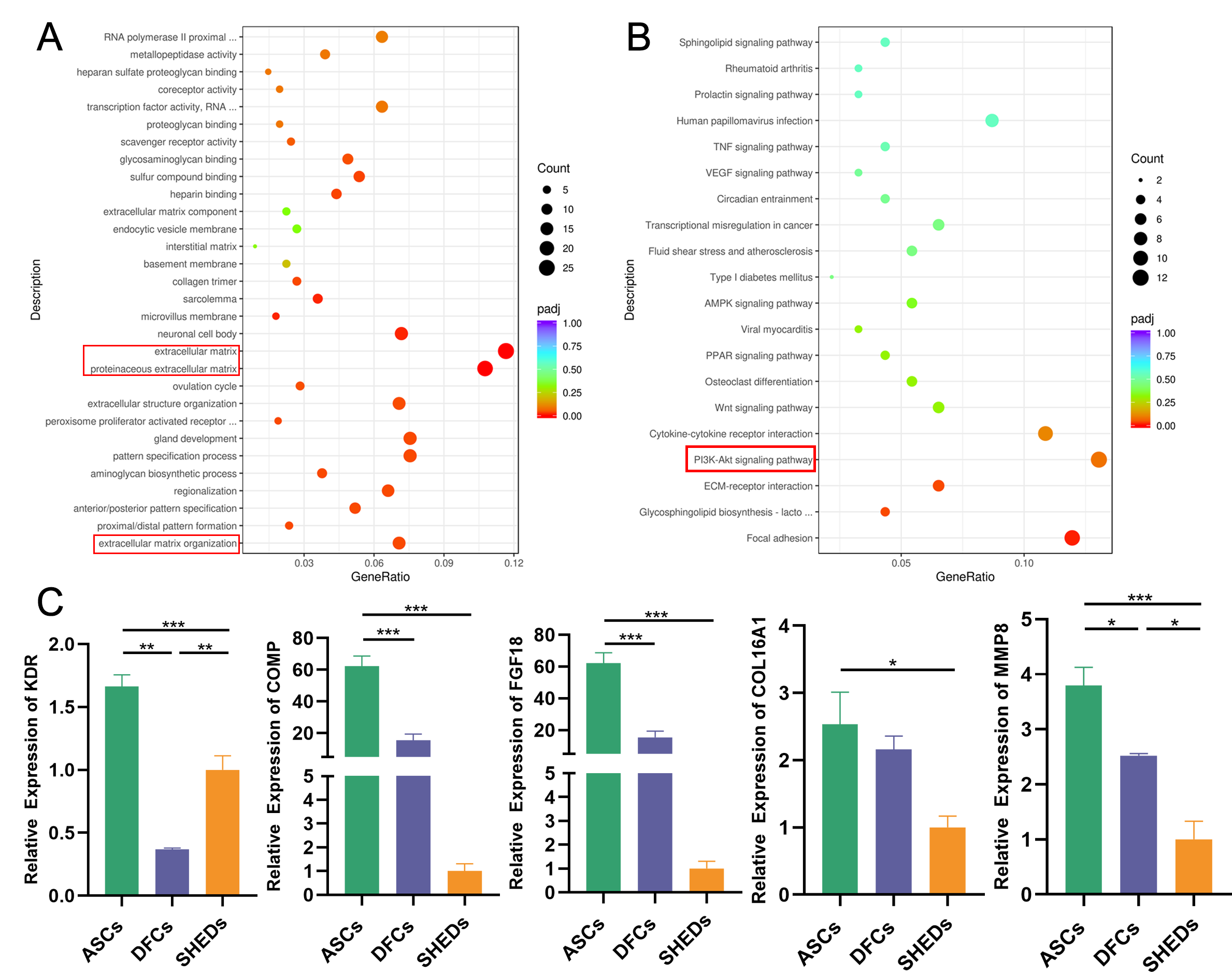

Supplement: Supplementary file 4 — Additional file 4. Fig. S3. Enriched terms related to the genes in ASCs/pTDM complex. (A) GO analysis showed that upregulated genes in ASCs/pTDM complex were significantly enriched in the extracellular matrix marked with a red box. (B) KEGG analysis showed that genes upregulated in ASCs/pTDM complex were significantly enriched in PI3K-Akt, focal adhesion, and cytokine-cytokine receptor interaction pathways. The PI3K-Akt pathway was marked with a red box. (C) mRNA expression levels of genes implicated in extracellular matrix and PI3K-Akt signaling pathway in cell/pTDM complexes (ASCs/pTDM, DFCs/pTDM, and SHEDs/pTDM) cultured for 4 days in vitro as indicated by RT-qPCR analysis. *p < 0.05, **p < 0.01, ***p < 0.001. [file 13287_2022_2907_MOESM4_ESM.tif]
